# The Aquatic Invertebrate *Hydra vulgaris* Releases Molecular Messages Through Extracellular Vesicles

**DOI:** 10.3389/fcell.2021.788117

**Published:** 2021-12-20

**Authors:** Maria Moros, Eugenio Fergola, Valentina Marchesano, Margherita Mutarelli, Giuseppina Tommasini, Beata Miedziak, Giuliana Palumbo, Alfredo Ambrosone, Angela Tino, Claudia Tortiglione

**Affiliations:** ^1^ Instituto de Nanociencia y Materiales de Aragón(INMA), CSIC-Universidad de Zaragoza, Zaragoza, Spain; ^2^ Istituto di Scienze Applicate e Sistemi Intelligenti “E. Caianiello”, Consiglio Nazionale Delle Ricerche, Pozzuoli, Italy

**Keywords:** extracellular vesicles, cell-cell communication, exosome, RNA-seq, *Hydra vulgaris*, aquatic invertebrate model

## Abstract

Recent body of evidence demonstrates that extracellular vesicles (EVs) represent the first language of cell-cell communication emerged during evolution. In aquatic environments, transferring signals between cells by EVs offers protection against degradation, allowing delivering of chemical information in high local concentrations to the target cells. The packaging of multiple signals, including those of hydrophobic nature, ensures target cells to receive the same EV-conveyed messages, and the coordination of a variety of physiological processes across cells of a single organisms, or at the population level, i.e., mediating the population’s response to changing environmental conditions. Here, we purified EVs from the medium of the freshwater invertebrate *Hydra vulgaris*, and the molecular profiling by proteomic and transcriptomic analyses revealed multiple markers of the exosome EV subtype, from structural proteins to stress induced messages promoting cell survival. Moreover, positive and negative regulators of the Wnt/β-catenin signaling pathway, the major developmental pathway acting in body axial patterning, were identified. Functional analysis on amputated polyps revealed EV ability to modulate both head and foot regeneration, suggesting bioactivity of the EV cargo and opening new perspectives on the mechanisms of developmental signalling. Our results open the path to unravel EV biogenesis and function in all cnidarian species, tracing back the origin of the cell-cell, cross-species or cross-kingdom communication in aquatic ecosystems.

## Introduction

Cell-cell communication is a mandatory condition for all the multicellular organisms as we know them; the possibility to exchange multiple and different signals between cells, at great distances or even between different organisms, supports the organism homeostasis and allows interacting with the surrounding environment. Intercellular cross-talk is achieved by various means, with the most straightforward one being the direct contact between communicating cells, also known as the juxtacrine signalling. Gap junctions are a well-known example of this form of communication and their functional characterization has been widely documented both in vertebrates and invertebrates (Takaku et al., 2014; Misu et al., 2016; Zong et al., 2016). Conversely, paracrine or endocrine signalling, namely the release and subsequent uptake of signalling factors, does not require a direct contact between donor and recipient cells, allowing for short- and long-distance intercellular communication. In addition to communication routes based on the secretion of soluble molecules in the extracellular space, the discovery of signalling factors embedded in bilayer lipid membrane structures, termed extracellular vesicles (EVs), has gradually expanded our view on how cellular cross-talk works. Despite the lack of general consensus about their classification and nomenclature (Thery et al., 2018) EVs are broadly divided into three main categories: apoptotic bodies, microvesicles and exosomes, mainly based on their different diameter and biogenesis route (Kalra et al., 2012; Veziroglu and Mias, 2020). First discovered in sheep reticulocyte cultures (Pan and Johnstone, 1983)^,^ (Harding et al., 1984) (Johnstone et al., 1987), exosomes are spheroidal nano-sized vesicles (30–150 nm) which originate during early-to-late endosome maturation. Multiple events of inward budding along the endosome membrane selectively gather proteins and nucleic acids from the cytosol into intraluminal vesicles (ILV). The late endosome is henceforth termed multivesicular body (MVB) and its fusion with the cell plasma membrane releases these vesicles into the extracellular space (Colombo et al., 2014; Kalluri and Lebleu, 2020; Skryabin et al., 2020). The protein content of exosomes partly reflects the biogenesis and maturation pathways that these vesicles undertake; consequently, specific proteins can be used as biochemical markers in order to confirm the presence of such vesicles in the isolated fractions. A fair number of tetraspanin transmembrane proteins, such as CD9, CD63 and CD81, are enriched in exosomes, reflecting the spatial reorganization that endosomal proteins go through before the formation of the MVB (Kowal et al., 2016; Anand et al., 2019). Similarly, lysosomal-associated membrane proteins LAMP1 and LAMP2 are late endosomal markers and can usually be found in exosomes (Colombo et al., 2014). However, different populations of exosomes may have distinct molecular signatures (Kowal et al., 2016; Juan and Furthauer, 2018). In fact, one fascinating feature of this type of EVs is that their content, comprising proteins, lipids and nucleic acids, is determined not only by the cell type they originate from, but also by the physiological state of the organism, resulting in major differences of these vesicles and their cargo in terms of quantity and quality (Ludwig et al., 2019). Such diversity is in agreement with the many processes exosomes have been implicated into, such as parasitic host-pathogen interaction (Coakley et al., 2015), viral infection, immune response, cardiovascular diseases, central nervous system disease, and cancer progression, shuttling into the receiving cells nucleic acids, metabolites, lipid and proteins (Kalluri and Lebleu, 2020). Of particular interest in cancer biology is their role in the “education” of distant cells in order to create a pre-metastatic niche, thus promoting metastasis (Peinado et al., 2012; Ghajar et al., 2013; Peinado et al., 2017). The intrinsic properties of exosomes in regulating complex intracellular pathways offer a new paradigm for their application as disease biomarkers, or as nanocarriers of therapeutic agents in cell-free therapies (Lu and Huang, 2020).

The wide portfolio of possible applications makes even more appealing the evolutionary study of EVs in model organisms, to increase our understanding in how they work and how they exert their functions in different contexts and environments. For instance, exosomes derived from *Drosophila melanogaster* have been identified as partly responsible for the establishment of Hedgehog (HH) and Wingless morphogenic gradients during the wing imaginal disc development (Gross et al., 2012; Gradilla et al., 2014). In *Caenorhabditis elegans* HH-related proteins are released on apical exosomes in a MVB-dependent manner involving V0-ATPase and P-4 ATPase activities (Liegeois et al., 2006; Wehman et al., 2011). However, very little is known about the role of EVs in aquatic environment. By virtue of their ability to transport abundant biological signals, to protect them from the highly heterogeneous and possibly damaging environment, and to increase the local concentration of one or more co-acting biomolecules that reach the target cells, EVs can have a profound impact on aquatic community structure and trophic level-interactions (Schatz and Vardi, 2018), providing important clues on interspecies and interkingdom relationships. Due to the conservation of key physiological mechanisms throughout the animal kingdom, evolutionary approaches to understanding EV biogenesis and bioactivity may profoundly impact on both fundamental and applied biology in vertebrates.

**FIGURE 1:**
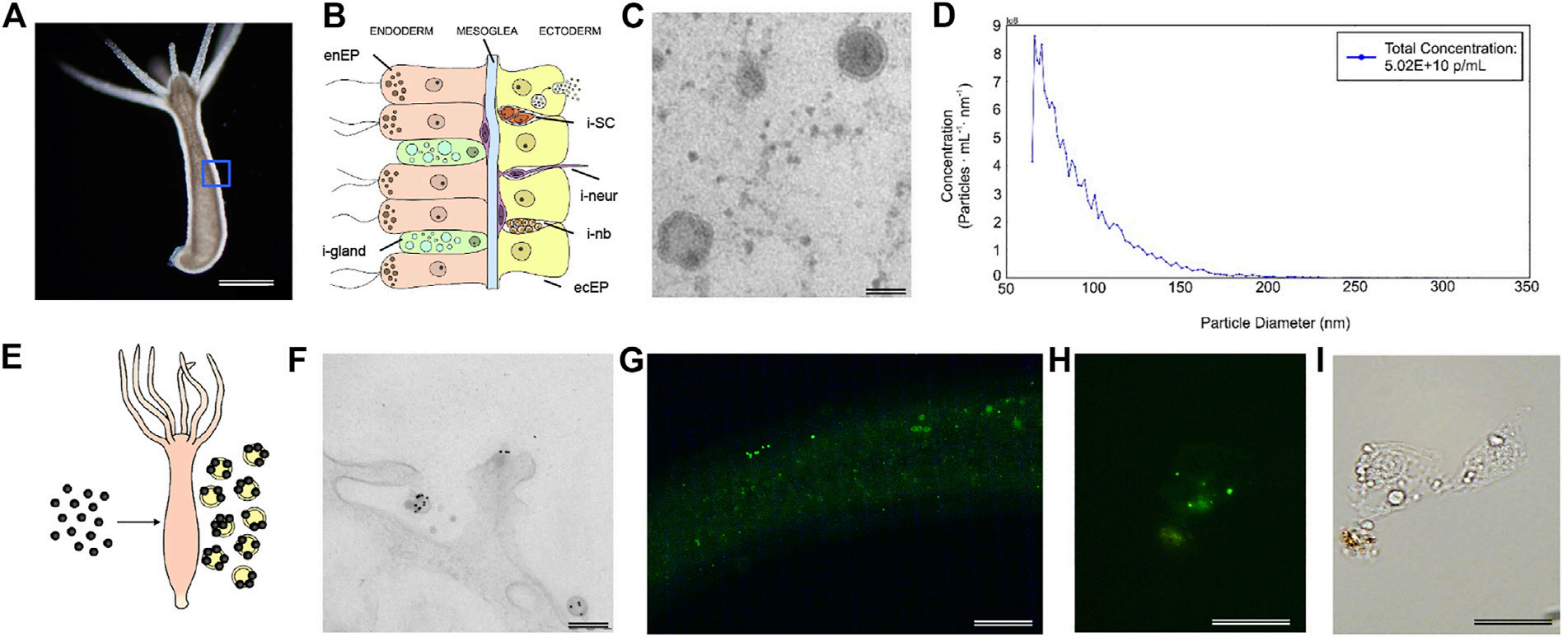
Morphological characterization of *Hydra* EV. **(A)** Dark-field image of a living *Hydra vulgaris* showing the simple body plan and **(B)** a schematic representation of the diploblastic tissue organization in a cross-section view. Epitheliomuscular cells (ecEP, yellow) face the outer medium and the inner cavity (enEP, pink). Interstitial stem cells (iSC, orange) lay interspersed into the ectoderm giving rise to a few differentiated cell types such as nematoblasts (i-nb), neurons (i-neur) and gland cells (i-gland). EV release into the medium is shown for a single ectodermal cell. **(C)** TEM image of isolated vesicles exhibiting round morphology and a bilayered membrane. Scale bar, 50 nm. **(D)** MRPS measurements show the distribution of the EV size, presenting two major peaks at 66 and 70.5 nm (using a TS-400 cartridge). **(E)** Schematic illustration of *in vivo* assembling of AuNP decorated EVs, by treatment of *Hydra* with AuNPs. **(F)** TEM analysis 48 h post treatment shows AuNPs on the surface of EVs. Scale bar 200 nm. **(G)** PKH67 labelled-EVs were incubated 2 h with living *Hydra*. The picture shows the body column with green fluorescent granular signals, clearly detectable also on fixed single cells **(H)** obtained by maceration of treated polyps. **(I)** Bright field image of the same cell. Scale bar 200 μm in **(G)**, 20 μm in **(H,I)**.

In this paper we report for the first time the presence of EVs in the freshwater cnidarian polyp *Hydra vulgaris* and characterized main morphological, biochemical and functional features. Classically used as a model organism in developmental biology, the structural simplicity of the *Hydra* body offers many advantages for a variety of investigations, from environmental ecotoxicology to nanobiotechnology, allowing to study the impact of exogenous nanostructured compounds (Quinn et al., 2012; Ambrosone et al., 2017; Tortiglione et al., 2017; Allocca et al., 2019) together with intracellular biotransformations and molecular responses (Veronesi et al., 2019; Moros et al., 2020a; Moros et al., 2020b) at animal, cell and molecular levels. The body wall is organized as a hollow tube, with an apical mouth opening surrounded by a ring of tentacles and a foot to anchor to a substrate. It is structured as a bilayer of epithelia, an ectoderm with epitheliomuscular cells facing the outer medium and an inner endoderm with all cells facing the body cavity, separated by an acellular mesoglea (Figures 1A,B). This tissue-like organization, with no organs and body fluids, is maintained by the continuous cell self-renewal and differentiation of three distinct stem cell lineages of endoderm, ectoderm, and interstitial stem cells, giving rise to a few differentiated cell type, i.e., neurons, gland cells, nematocytes and gametes (Bosch, 2009; Hobmayer et al., 2012). The plasticity and remodelling capacity of the body reaches the highest expression during the regeneration process (Steele, 2002; Bode, 2003; Bosch, 2009; Vogg et al., 2019b). Indeed, the ability to regrow the whole body from tissue pieces or even cell aggregates has made Hydra a one-of-a-kind platform to study regeneration processes and to shed some light on why some organisms have partially lost this ability (Li et al., 2015). The Wnt/βcatenin signalling pathway represents the major developmental pathway acting in adult *Hydra* to maintain the oro-aboral axis, and it is reactivated during injury and throughout the regeneration to rebuild missing structures from amputated polyps (Hobmayer et al., 2000; Chera et al., 2009b; Lengfeld et al., 2009). Functional and transcriptomic evidences have recently showed the early activation of the Wnt/βcatenin signalling pathway as part of a generic transcriptional response to injury, which initiates an oral patterning cascade independent from the tissue context (Petersen et al., 2015; Cazet et al., 2021). The outcome of this activation appears regulated by long range signals generated by tissue organizers located at both animal poles (Bode, 2009; Petersen et al., 2015; Gufler et al., 2018; Wenger et al., 2019; Cazet et al., 2021), whose existence was proposed 40 years ago, by means of classical tissue manipulation techniques (Macwilliams, 1983). To date, in *Hydra*, no vehicles have been isolated for lipid-modified and poorly soluble signalling molecules. The EV function in developmental signalling, promoting the release and spread of canonical ligands and/or mediating the transfer of a variety of biologically active molecules from one cell to another is well documented (Gross et al., 2012; Zhang and Wrana, 2014; Mcgough and Vincent, 2016). This prompted us to search for similar EV mediated signalling mechanisms also in *Hydra*, and to shed light on the early evolution of signalling mechanisms in metazoans. We developed a strategy for EV purification from the polyp culture medium, characterized their morphology and their molecular content at protein and RNA levels, and provide evidence on the bioactivity of their cargo. The shuttling of EV messages in target cells shows that this mechanism of cell-cell communication evolved early in animal evolution and may mediate not only physiological processes but also cross-species or cross-kingdom communication in aquatic ecosystems.

## Materials and Methods

### Animal Culture

*H. vulgaris* (strain Zurich) was asexually cultured in Hydra medium (HM: 1 mM CaCl_2_ and 0.1 mM NaHCO_3_) at pH 7. The animals were kept at 18 ± 1°C and fed thrice a week with freshly hatched *Artemia salina* nauplii.

### Collection of Extracellular Vesicles

For EV collection, 250 polyps were placed in 10 ml of HM in 6 cm Petri dishes. After 4 days starvation, Hydra conditioned medium was collected and centrifuged using a Beckman Coulter Avanti J-25 centrifuge (rotor JA-25.15) at 10,000 g for 30 min to remove tissue patches and at 20,000 g for 30 min to pellet any cellular debris. The resulting supernatant was then centrifuged at 74,200 g for 150 min at 4°C and the pelleted EVs were washed in HM followed by one last centrifugation. Pelleted EVs were resuspended in either PBS or Hydra medium.

### Transmission Electron Microscopy

EVs were visualized using TEM following a slightly modified protocol to that reported by Thery et al. (2006). Briefly, EVs were fixed with an equal volume of 4% paraformaldehyde and deposited onto a Formvar-carbon coated electron microscopy grid. Once dried, the grids were washed with PBS, fixed with 1% glutaraldehyde for 5 min and washed with distilled water 8 times. The samples were stained with 1% ammonium molybdate for 2 min, dried and observed using a FEI Tecnai 12,120 kV or JEOL United States, Inc., United States) at 80 kV. *Hydra* polyps were dissected into appropriate pieces to fit into cup-shaped HPF specimen carriers. Tissue pieces were pipetted into a 0.2- or 0.3-mm deep carrier and covered with an additional carrier. Finally, the obtained sandwiches were cryo-immobilized by HPF. Frozen sandwiches were transferred into appropriate containers for storage in Liquid N_2_ for later use or subjected to freeze substitution (FS). The frozen samples were transferred under liquid N_2_ into cryo-vials containing frozen FS cocktails (anhydrous acetone plus 1% OsO_4_ and 0.1–0.2% uranyl acetate). Subsequently, the lids were screwed loosely onto the vials to permit safe evaporation of excess N_2_ gas. The vials were placed into the precooled FS device and after about 1 h the lids tightened and FS was performed for at least 8 h at −80 to −90°C; warming up to −55°C at a rate of 5–10°C per hour, subsequent post-fixation and staining at −55°C for 6 h, followed by warming up to −30°C at a rate of 5–10°C per hour where samples were left for an additional 3 h. Finally, samples were washed three times with acetone (10 min each), 16 h in 10% epoxy resin in acetone, 6 h in 30% epoxy resin in acetone, 16 h 70% epoxy resin in acetone, 6 h 100% epoxy resin in acetone, 16 h 100% epoxy resin in acetone. Then the samples were placed in fresh resin and placed in oven at 60°C overnight.

Sample serial thin (70 nm) section of animals were cut with a diamond knife and mounted onto 150–200 mesh Hex grids. Ultrathin sections were examined with transmission EM Libra 120 EFTEM (Zeiss, Oberkochen, Germany) at 80 eV.

### EV Sizing and Counting

Microfluidic Resistive Pulse Sensing (MRPS) (Cimorelli et al., 2021): measurements of isolated extracellular vesicle size and concentration were performed using the nCS1™ instrument (Spectradyne LLC, United States), which is based on the microfluidic resistive pulse sensing technology. EVs isolated from 250 polyps and resuspended in Hydra medium were diluted in equal volume with Tween/PBS buffer in order to meet the instrument requirements regarding medium conductivity. EV samples were analysed with TS-400 and TS-300 cartridges (measurement range 60–400 nm and 50–300 nm, respectively).

Dynamic light scattering (DLS) was performed with a Malvern Zetasizer using EVs diluted in PBS at pH 7.4. Three runs were made at 25°C and at 90°scattering angle.

### Western Blot Analysis

Protein concentration was determined using a colorimetric assay based on the Bradford Assay (ThermoFisher) and samples were diluted with PBS to obtain equal protein concentration (EV and whole *Hydra* homogenates). Samples were mixed with 4x Laemmli Sample Buffer containing 2-mercaptoethanol (Biorad) and boiled for 5 min at 95°C. Proteins were resolved by SDS-PAGE (mini protean TGX precast any KDa gel (Biorad)), blotted onto PVDF membranes (Amersham) and blocked o/n at 4°C in 5% non-fat dry milk in PBS-T (0.5% Tween-20). Membranes were then incubated with the primary antibody for 2 h at room temperature, washed with PBS-T 0.1%, and incubated with goat anti-rabbit IgG Antibody, (H + L) HRP-conjugated secondary antibody (1:4,000, BioRad) for 1 h at room temperature. Finally, protein bands were detected using an enhanced chemiluminescence reagent (Clarity ECL Substrate (Biorad)) and imaged with ChemiDoc XRS *System* (BioRad) using Image Lab software (BioRad). Antibodies: rabbit anti-Hydra Annexin B12 1:1,000 (kindly gifted by Dr. M. Isas, Keck School of Medicine of USC Los Angeles, CA), mouse anti-β-Actin−Peroxidase (1:40,000, Sigma).

### EV Staining and Uptake

EVs obtained as described before were labelled with PKH67 Green Fluorescent Cell Linker (Sigma Aldrich) following manufacturer’s protocol, with minor modifications. Briefly, EVs obtained from 500 polyps in HM were added to 1 ml of diluent C. As a negative control, 1 ml of diluent C was mixed with the same volume of HM. Afterwards, 6 μL of PKH67 dye was added and mixed for 30 sec by gently pipetting. After 5 min incubation at room temperature, 2 ml of 10% BSA was added to quench the unbound dye. EVs were washed and isolated by ultracentrifugation as described before. *Hydra* were incubated with PKH67- labelled EVs and with the negative control up to 5 h, washed with HM and imaged under a fluorescence microscope (Zeiss).

### Regeneration Experiments

All the experiments were carried out using adult polyps starved for 24 h. For the head regeneration experiments, batches of 25 budless polyps were bisected at 80% body length (sub-hypostomal cut) or 50% body length (mid-gastric cut) and left regenerating for up to 72 h in presence of EVs isolated from 250 polyps (100 µL) in a total volume of 300 µL. The regeneration process was monitored at different time points using an optical microscope and polyps were grouped into four stages according to their tentacle morphogenetic features.

For the foot regeneration experiments, groups of 15 animals were bisected at 20% body length and transferred in HM containing EVs isolated from 250 polyps (100 µL) in a total volume of 300 µL. After 24 h, polyps were relaxed in 2% urethane in HM for 2 min and pre-fixed by adding 4% PFA/HM for 15 min. After removing the pre-fixation medium, animals were fixed for 1–3 h in 4% PFA/HM at 18°C and washed three times in PBS to remove any residual fixative. For the peroxidase assay, 2 ml of DAB solution was prepared according to the manufacturer’s instructions (Sigma-Aldrich tablets) and used for 15 min on the fixed animals. In order to stop the colorimetric reaction, the polyps were washed five times with ddH_2_O and then mounted with PBS/Glycerol 1:1 on microscope slides for imaging.

Stained animals were observed by an inverted microscope (Axiovert 100, Zeiss) and images of each foot were taken with a digital camera (Olympus, DP 70) under the same conditions of acquisition (light and exposure time). The colorimetric analysis was performed using the HSB format on the acquired images in the ImageJ software version 1.53e. Three staining categories were arbitrarily set according to DAB positive area extension as low (0–9,000 μm^2^), medium (9,000–19,000 μm^2^) and high (19,000–28,000 μm^2^).

### EV Pre-treatment With Proteinase and RNAse

Prior to total RNA extraction, EVs isolated by ultracentrifugation were enzymatically treated to eliminate the free RNA derived from the ribonucleoprotein complexes that could be present in the conditioned medium. Isolated vesicles were treated with 1 mg/ml of proteinase K for 30 min at 37°C. Afterward, the samples were incubated 10 min at 65°C to inactivate the proteinase K and then treated with 10 µg/ml of RNAse A for 15 min at 37°C.

### qRT-PCR Analysis

After treating polyps for 24 and 48 h with EVs isolated from 750 *Hydra*, total RNA was extracted from animals using Trizol or from EVs using Total RNA Extraction Kit (Norgen Biotek, Corp.) following manufacturer’s instructions and its concentration was determined using a SmartSpec plus spectrophotometer (Biorad). The first-strand cDNA was synthesized by High Capacity cDNA Reverse Transcription Kit (Applied Biosystem). qRT-PCR was performed using a Fast SYBR^TM^ Green Master Mix with premixed ROX (Applied Biosystems), in a StepOne Real-Time PCR System (Applied Biosystem) under the following fast cycling steps: initial denaturation for 20 s at 95°C, followed by 40 cycles at 95°C for 3 s, 60°C for 30 s. In addition, melting curves (20 min, from 59° to 90°C) were generated to check any undesired amplification products. *EF1α* was used as internal control.

In order to validate RNA-seq analysis, a presence/absence experiment of selected targets was performed using an EV sample isolated from 2000 polyps. Specific primers of *Hydra* genes *Wnt3a, β-catenin, actin and Hsp70* were designed using the Primer3 software (http://frodo.wi.mit.edu/primer3/) and are listed in Supplementary Table S3, together with the corresponding GenBank accession numbers. Due to the qualitative nature of the presence/absence analysis, no internal control was used for the amplification reaction. The presence of such transcripts in the EV sample was confirmed by their cDNA amplification compared to negative controls, where no amplification occurred. Additional analysis by generation of melting curves (from 60 to 95, 0.5°C increment) confirmed sequence specific amplification in EVs and the absence of amplification in No Template Control (NTC). Mean C_t_ values for each gene and its NTC are shown in Supplementary Table S2

### RNA Seq

EVs were collected at different times and stored at −80°C. Total RNA extraction was performed on EV samples collected from 10,000 polyps overall and in two replicates using “Total RNA Purification Kit” (Norgen Biotek Corp.) and following the manufacturer’s supplementary protocol for exosomal RNA purification, with one modification: the optional DNA removal step was carried out using RQ1 RNase-Free DNAse (Promega) at 0.1 U/mL. The two samples were sent to the Sequentia Biotech S.L. (Barcelona, Spain) company for quality check, cDNA library preparation using “Ovation® SoLo RNA-Seq Systems” (NuGEN Technologies, Inc.), and sequencing via a paired-end chemistry on an Illumina platform. After quality inspection of the produced reads with fastqc (https://www.bioinformatics.babraham.ac.uk/projects/trim_galore), reads were cleaned to remove the first five bases of read1, the Illumina adapter sequence “AGATCGGAAGAGC” and the bases with quality below 20 from the 3′ end using the program TrimGalore! (https://www.bioinformatics.babraham.ac.uk/projects/trim_galore); the cleaned reads shorter than 35 bp were removed from further analysis (9.3% of the produced read pairs). Trimmed reads were then aligned using the program STAR (version 2.7.1a)^78^ against the NCBI *Hydra vulgaris* reference genome (assembly Hydra_RP_1.0), while for gene expression quantification, trimmed reads were also aligned on the corresponding transcript sequences GCF_000004095.1_Hydra_RP_1.0_rna.fna.gz using the program kallisto (Dobin et al., 2013; Bray et al., 2016). Kallisto was also used to quantify genes using the reference transcriptome used in Siebert et al. (2019) in order to directly map the genes with the cell types where they were identified.

### Protein Identification by MALDI-TOF/TOF

To perform the MALDI-TOF/TOF EV samples were first run into a SDS-Page gel. EV total proteins were quantified using a Bradford assay, 4x Laemmli Sample Buffer with 2-mercaptoethanol (Biorad) was added, samples were heated to 95°C for 5 min and they were run in a mini protean TGX precast 7.5% gel (Biorad). The gel was silver stained following standard procedures.

In-gel digestion- Protein bands were manually excised with a cutter, and in-gel digested with an automatic digestor (Intavis, Bioanalytical Instruments, Cologne, Germany). Briefly, spots were washed with water, ammonium bicarbonate (100 mM), and acetonitrile. Next, samples were reduced by incubation with DTT (10 mM) at 60°C for 45 min and alkylated by incubation with iodoacetamide (50 mM) at room temperature for 30 min. Finally, proteins were digested with trypsin overnight at 37°C (5 ng/μL, Trypsin Gold, Promega, WI, United States). Digestion was stopped by adding 0.5% TFA (trifluoroacetic acid), and tryptic peptides were extracted sequentially with increasing concentrations of acetonitrile in water. Peptides were concentrated and desalted by passing it through ZipTip C18 tips (Millipore) following the manufacturer’s instructions and eluted with 50%ACN 0.1%TFA.

Peptides were spotted into an Opti-Tof 384 well insert plate (Sciex) with a saturated solution of alpha-cyano-4-hydroxycinnamic acid (CHCA) prepared in 50% ACN/0.1% TFA. Proteins were identified using a 4800plus MALDI-TOF/TOF (Sciex) in the reflector mode with accelerating voltage of 20 kV, mass range of 800–4000 Da and 1,000 shots/spectrum. MS/MS spectra were acquired automatically on the 20 most intense precursors. Spectra were calibrated externally using a standard protein mixture (4,700 Calmix, ABSciex).

### Protein Identification

Proteins were identified with the search engine Mascot (Matrix Science, London, United Kingdom) against the NCBI prot database of *Hydra* (29,584 sequences). Search parameters used were: missed cleavage 1, fixed modifications carbamidomethyl (cysteines), variable modifications oxidation (methionine) and peptide and fragment mass tolerance 0.2 and 0.3 Da, respectively. Proteins with a score above 23 were considered a positive hit.

### Protein Identification by LC-ESI-MS/MS

In solution digestion**-** Samples were evaporated and resuspended in 10 μL of denaturing buffer (6 M urea, 100 mM Tris buffer pH 7.8). Next, cysteines were reduced with 1.5 μL DTT (200 mM) for 30 min at 37°C and alkylated with 6 μL of iodoacetamide (200 mM) for 30 min in the dark. Unreacted iodoacetamide was consumed adding 6 μL of the reducing agent (200 mM DTT) for 30 min at room temperature. Samples were diluted with 50 mM ammonium bicarbonate to a final concentration less than 1 M of urea. Digestion was carried out overnight with the enzyme trypsin (Gold Trypsin, Promega) at 37°C and a 1:10 ratio (enzyme/protein). Reaction was stopped adding concentrated formic acid (Sigma). Samples were evaporated, resuspended in 2% acetonitrile, 0.1% formic acid and filtered through 0.45 μm filters. Protein identification was performed on a nano-LC system (Tempo MDLC, Eksigent, Dublin, CA, United States) coupled to a hybrid triple quadrupole/linear ion trap mass spectrometer (4000 QTRAP, Sciex). After precolumn desalting, tryptic digests (1 μg) were separated on a C18 column (Acclaim PepMap100, 75 μm id, 15 cm, 3 μm particle size, Thermo Scientific, United States) at a flow rate of 300 nL/min and a 120 min linear gradient from 5 to 35% ACN in 0.1% formic acid. The mass spectrometer was interfaced with a nanospray source equipped with uncoated fused silica emitter tip (20 μm inner diameter, 10 μm tip, NewObjective, Woburn, MA) and was operated in the positive ion mode. MS source parameters were as follows: capillary voltage 2800 V, source temperature 150°C, declustering potential (DP) 85 V, curtain and ion source gas (nitrogen) 20 psi, and collision gas (nitrogen) high. Analyses were performed using an information dependent acquisition (IDA) method with the following steps: single enhanced mass spectra (EMS, 400–1,400 m/z) from which the eight most intense peaks were subjected to an enhanced product ion [EPI (MS/MS)] scan. Proteins were identified with the search engine Mascot (v2.3 MatrixScience, United Kingdom) using NCBIprot database of *Hydra* (29,584 sequences). Search parameters used were: missed cleavages 1, fixed modifications carbamidomethyl (cysteines), variable modification oxidation (methionine) and peptide and fragment mass tolerance 0.5 and 0.3 Da, respectively. Only proteins with an overall Mascot score higher than 32 and at least two matching spectra were considered in the final list.

## Results

### *Hydra vulgaris* Releases Round-Shaped Extracellular Vesicles Into the Aquatic Environment

EVs were isolated from the medium of starved polyps by differential centrifugation, following a well-established protocol (C Thery et al., 2006), slightly modified. Pelleted EVs suspended in Phosphate buffered saline (PBS) where then characterized by Transmission Electron Microscopy (TEM). Figure 1C shows the round shape of isolated EV and the presence of double membrane around the electron dense vesicle (Supplementary Figure S1). By microfluidic resistive pulse sensing (MRPS) a yield of 5 × 10^10^ particles/mL was estimated from a typical preparation (250 polyps into 10 ml of medium), while the distribution of the particle size showed two peaks at 66 and 70.5 nm (Figure 1D and Supplementary Figure S1). A good correlation with these measurements was obtained through other approaches, such as Dynamic Light Scattering (DLS) analysis and TEM (Supplementary Figure S1), both confirming the average size of 66 nm, and suggesting an enrichment in exosome-like vesicles. The presence of EVs on *Hydra* ectodermal cell surface was previously supposed during an ultrastructural study describing several inward and outward mechanisms negotiating the entrance, trafficking, and clearance of gold nanoparticles in animal tissues (Marchesano et al., 2013). To confirm this evidence, *Hydra* polyps were pulsed (24 h) with positively charged AuNPs, synthesised as previously described (Marchesano et al., 2013) and 48 h later processed for TEM (Supplementary Figure S2). Semi-thin slices showed the presence of round nanovesicles decorated with AuNPs on the external membrane of ectodermal cells, laying into the glycocalix (Figures 1E,F), confirming *in vivo* assembly of AuNP-EV structures as part of the secretory route (Marchesano et al., 2013). This assembly demonstrates a strong interaction between EV membranes and positively charged AuNPs, likely due to electrostatic interaction.

The ability of EVs to be internalized into *Hydra* tissues was investigated by labelling freshly isolated EVs with PKH67 and incubating living polyps up to 5 h. Distinct green fluorescent signals were found on the body column and tentacles 2 h post incubation and in single cells obtained by maceration of treated polyps, indicating an efficient uptake of EVs into *Hydra* cells (Figures 1G–I; Supplementary Figure S3). These signals became hardly detectable after 5 h, possibly suggesting the processing of the EVs into the recipient cells.

### Hydra EVs Shuttle Typical Exosome-Associated Proteins and Key Components of Axial Patterning

Initially, a MALDI-TOF/TOF analysis performed on EV protein bands excised from an SDS-PAGE gel identified Annexin B12 and actin, whose presence was confirmed by immunoblot analyses (Supplementary Figure S4). Then a large scale EV isolation was achieved from 8,000 *Hydra*, and protein extracts were used for LC-ESI-MS/MS analysis and protein identification. The analysis from two biological replicates identified 52 proteins with a Mascot score >32 (Supplementary Table S1). Table 1 shows selected unique *Hydra* proteins grouped for function. Remarkably, 29 proteins were present also in the Exocarta protein database (http://www.exocarta.org/), suggesting the exosome–like nature of the isolated EVs. The exosome-specific proteins include two annexins, a synthenin1-like protein involved in the biogenesis of exosomes, a programmed cell death 6-interacting protein involved in the MVB formation and the CD151 antigen belonging to the Tetraspanin family. Among others, chaperones (Heat Shock Proteins), cytoskeletal proteins (Actin, Tubulin), extracellular matrix constituents (Fibrillin 1, Fibrillin 2), and important components of cell-cell adhesion (Protocadherin fat 4) and vesicle biogenesis (Synthenin, Programmed cell death 6-interacting protein, Polyubiquitin-B) were found. Altogether the variety of putative proteins identified in the *Hydra* EVs, either structural or involved in cell-cell communication and signal transduction, suggests multiple functional roles played by EVs in adult *Hydra*, and their correlation with known exosome proteomes.

**TABLE 1:** Name and putative function of selected proteins identified by ESI-MS/MS analysis.

Accession No.	Protein name	Putative function	Matched peaks	Matched peptide	Mascot score	ExoCarta
**Cytoskeletal proteins**						
CDG70628	Actin, cytoplasmic 1	Cytoskeleton component	18	16	235	Yes
XP_002161913	Tubulin beta chain	Cytoskeleton component	10	8	169	Yes
XP_004208788	Tubulin alpha-1D chain	Cytoskeleton component	5	4	41	Yes
**Extracellular matrix constituents**						
XP_012558692	Fibrillin-1	Microfibril assembly	11	10	125	Yes
XP_002166874	Fibrillin-2 isoform X1	Microfibril assembly	9	7	107	Yes
XP_002154154	Latent-transforming growth factor beta-binding protein 4-like	Extracellular matrix constituent	12	12	80	Yes
**Membrane proteins**						
NP_001296699	Annexin B12	Calcium-dependent membrane binding	7	4	100	No
XP_004212135	Protocadherin Fat 4, partial	Cell-cell adhesion	7	7	89	Yes
XP_004212674	Protocadherin Fat 1, partial	Cell-cell adhesion	17	17	71	Yes
XP_012553809	Annexin A4-like	Calcium-dependent membrane binding	14	13	61	Yes
XP_002165348	CD151 antigen	Exosome marker	5	5	56	Yes
XP_004211099	Usherin-like, partial	Integral component membrane	2	2	47	No
**Cell-cell communication and signal transduction**						
AAN87350	14-3-3 protein B	Growth adaptation to food supply	2	2	54	No
XP_002156827	Ras-like GTP-binding protein RHO	Signal transduction	2	2	52	Yes
AEW90237	Voltage-gated sodium channel Nav2.1, partial	Neuronal signaling in Hydra	15	15	34	No
XP_012554672	cAMP-dependent protein kinase type II regulatory subunit-like isoform X2	cAMP-mediated signaling pathway	5	4	42	Yes
XP_004209147	Cell division control protein 42 homolog	Small GTPase-mediated signal transduction	2	2	36	No
AAW82079	Thypedin	Foot formation stimulator	5	5	57	No
**Others**						
XP_002159321	Syntenin-1-like isoform X2	Biogenesis of exosomes	9	8	99	Yes
XP_002162060	Programmed cell death 6-interacting protein	Multivesicular body biogenesis	10	9	43	Yes
XP_002155023	Polyubiquitin-B	Vesicle cargo sorting	11	7	43	Yes
XP_002156816	Uncharacterized protein LOC100207118	Metalloproteinase with Thrombospondin motifs	5	5	130	—
CAJ33888	Putative serine protease inhibitor	Protective function against excessive autophagy	5	5	54	No

A comprehensive transcriptomic analysis was performed on total RNA from purified EVs (from two biological replicates) generating two cDNA libraries and conducting pair-end next-generation sequencings. The sequenced reads were first aligned against the NCBI *Hydra vulgaris* reference genome (assembly Hydra_RP_1.0), where a percentage of 32 and 22% could be mapped. For gene expression quantification, the reads were also directly aligned using the program kallisto on the Hydra_RP_1.0 transcript sequences, where the total mapped reads on transcripts were 50 and 45% of the reads mapped on the genomic sequence. The presence of a subset of transcripts was confirmed in EV-derived RNA by using reverse transcription polymerase chain reaction PCR (RT-PCR). The selection was based on manual screening of genes already identified in EVs and exosomes (*hsp70* and *actin*) *or* belonging to the Wnt/β−catenin signalling pathway (*β−catenin*, and *Wnt3*). We then used the minimum expression level in TPM (Transcripts Per Kilobase Million) of the validated genes to define an empirical detection threshold (0.4 TPM in at least one library). Using this definition, we selected 6,100 detected genes, among which 5,499 were protein-coding, 525 long non-coding and 31 tRNA genes. The detected genes were functionally annotated with the known associated Gene Ontology (GO) terms using OrthoDB to highlight the most important biological processes encoded by EV mRNAs (Kriventseva et al., 2019). The full list of the GO categorized genes is provided in Supplementary file 1. Interestingly, this analysis revealed that the most abundant transcripts within the Molecular Functions GO category are related to catalytic activity (alcohol dehydrogenase, sulfite oxidase), proteases and proteinase inhibitors (cathepsin, several members of the astacin family, antistasin), hydrolase and transferase activity. Within the Cellular Component GO category the genes encoding for intracellular and cytoplasmic anatomical structures were highly abundant, such as actin, cophylin, α-tubulin chain, β tubulin chain, talin-2, secreted glycoproteins (mucin 2-like, mucin 5AC-like), and LAMP, a well-known exosome marker. The transcripts categorized into the Biological processes GO class included the classes of protein metabolism (tRNA synthase, eIF3, eIF4, eEF1α, tRNAs), signal transduction pathways (G-protein coupled receptors, Rho family, guanidine nuclear exchange factors, Wnt3, gremlin) and response to stress (Hsp70, Hsp90, Superoxide dismutase, Glutamate dehydrogenase, thioredoxin-like).

Beside transcripts identification, lncRNA species were detected from the RNA-seq analysis. In one case the lncRNA was mapped adjacent to the *mucin5AC* gene locus, detected both at mRNA and protein levels, suggesting the possibility that EVs may shuttle all molecular components for immediate and late availability of key information in target cells. The association of lncRNA with exosomes was first demonstrated by the analysis of human plasma-derived exosomes by RNA-seq (Huang et al., 2013), and has been recently functionally characterized and linked to disease, including a list of cancers, where they act *via* epigenetic regulation of key target genes. Their presence in *Hydra* EV might suggest a regulatory function in the receiving cells.

**FIGURE 2:**
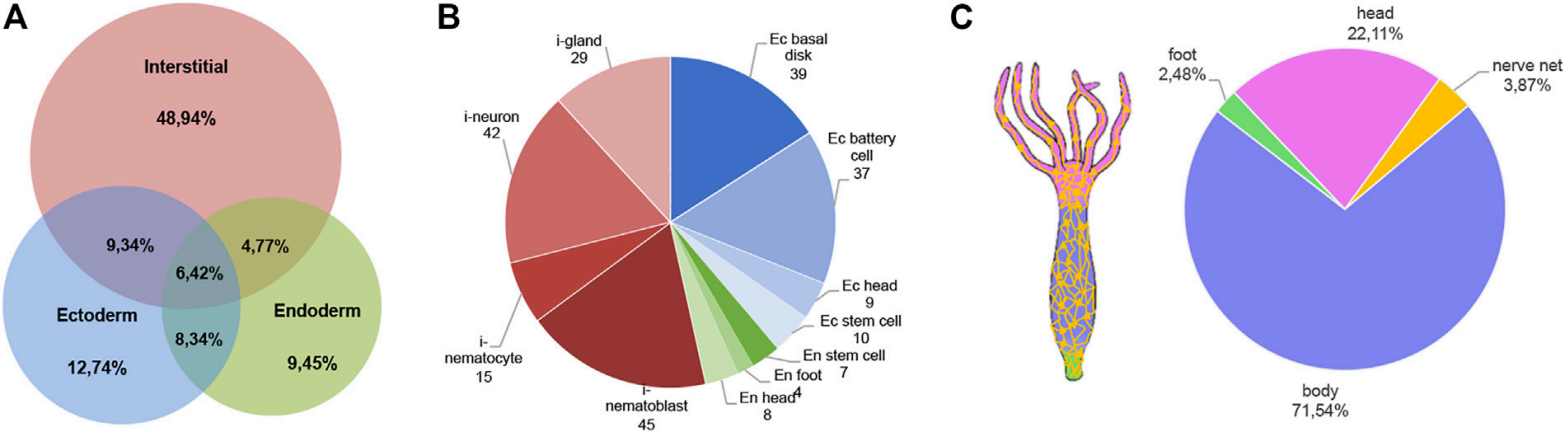
Molecular contribution of cell lineages to the *Hydra* EV transcriptome. **(A)** Venn Diagram showing cell lineage contribution to the EV transcriptome. The clusters of cells included into each cell lineage were identified by scRNA-seq analysis recently reported (Siebert et al., 2019) and are listed in Supplementary Figure S5. **(B)** Distribution of EV transcripts matching the scRNA-seq atlas, subclustered according to their unique presence in a specific cell type of ectoderm (blue tones), interstitial (red tones) and endodermal (green tone) cell lineage. Transcripts common to multiple cell lineages are not included. **(C)** Anatomical source of EV transcripts. The distribution shows EV transcripts derived from all body regions, including the nerve net.

In order to map the EV cellular source in the animal body we used a recently-published single-cell RNA-seq (scRNA-seq) atlas (Siebert et al., 2019) to match *Hydra* EV-associated genes with molecular signatures of specific cell clusters, which were grouped according to cell lineage (ectodermal, endodermal and interstitial cell lineage), or anatomical location (head, body column and foot) (Figure 2). An alignment rate of 12% was obtained and 4,997 transcripts were detected, of which 2,975 were found in at least one cell type in the scRNA annotated on Swiss-prot (TPM 0.2 in at least one library, Supplementary file 2). A large percentage of transcripts (48%) was clustered as specific of the interstitial cell lineage, while those derived from the ectodermal (12.7%) and endodermal cell lineage (9.45%) were less abundant. By subclustering the EV transcripts according to unique cell type expression, using the same cell categories and differentiation trajectories identified in the scRNA-seq (Siebert et al., 2019), a remarkable contribute of basal disk cells, battery cells, neurons and nematoblasts emerged (Figures 2B,C). A detailed analysis subclustering EV transcripts into cell types of each cell lineage is shown in Supplementary Figure S5. Overall, this indicates the mixture of precise molecular information from multiple cell types in *Hydra* EVs. The list of the five top genes in each cell cluster is provided in Supplementary Table S4 and the list of the 10 top genes identified matching the scRNAseq is shown in Supplementary Table S5. Interestingly, many genes belonging to the Wnt/β catenin signalling pathway were identified (Table 2), including *Wnt3*, *β−catenin*, *naked cuticle* (Petersen et al., 2015) together with some inhibitors such as *dkk1/2/4* (Augustin et al., 2006; Guder et al., 2006), *astacin* (Ziegler et al., 2021), *thrombospondin* (Lommel et al., 2018), and *sp5* (Vogg et al., 2019a). Remarkably, *dkk1/2/4* and *astacin* were found among the most abundant transcripts specific of zymogen and granular mucous gland cell classes, strongly indicating their role in restricting the head organizer activity in the hypostome. By comparing the EV transcriptome to the proteome many annotated proteins (found also on exosome proteomes) were identified also at transcript level (*actin, tubulin, annexinB12, hsp70*)*,* indicating the concomitant transfer of molecular information for immediate and programmed response in the target cells.

**TABLE 2:** List of EV transcripts involved in the Wnt/β-catenin signalling pathway matching the scRNA-seq database.

	Genes involved in Wnt/β-catenin signaling pathway					
Gene id	Gene id (NCBI)	Gene name	Wnt modulator	Lineage	Cell type	Anatomical region
t8678aep	100,192,274	Dickkopf 1/2/4-A	Negative regulator	Interstitial	i_zimogen gland cell	Body
t14102aep	100,192,275	dickkopf-like protein Dlp-1	Negative regulator	Interstitial	i_zimogen gland cell	Body
t1688aep	100,192,284	β-catenin	Positive regulator	Ectoderm, Interstitial	Ec_battery cell, i_neuron/gland_cell_progenitor	Tentacle, Body
t29291aep	101,237,470	transcription factor Sp5-like	Negative regulator	Ectoderm, Endoderm	Ec_battery cell, En_head, En_tentacle	Head
t11826aep	100,199,257	Tcf	Positive regulator	Endoderm, Interstitial	En_head, i_neuron_ec	Head, Body
t34763aep	100,205,238	Naked cuticle	Negative regulator	Endoderm	En_head	Head
t13357aep	100,199,272	β-catenin-like protein 1	Positive regulator	Interstitial	i_female germline1, i_male germline, i_nb1, i_neuron/gland cell progenitor, i_SC, i_stem cell/progenitor	Body
t14194aep	100,203,050	Wnt3	Positive regulator	Endoderm	En_head	Head
t18735aep	100,213,948	FoxA/Budhead	Positive regulator	Interstitial	i_neuron/gland_cell_progenitor	Body
t31094aep	100,215,335	Foxd2-like		Endoderm	En_foot	Foot
t16296aep	100,199,630	HAS-7	Negative regulator	Interstitial	i_granular mucous gland cell, i_zymogen gland cell	Head, Body
t474aep	100,214,250	HmTSP	Negative regulator	Endoderm	En_tent-nem (pd), En_tentacle	Tentacle

### EVs Modulate Head and Foot Regeneration in *Hydra*

**FIGURE 3:**
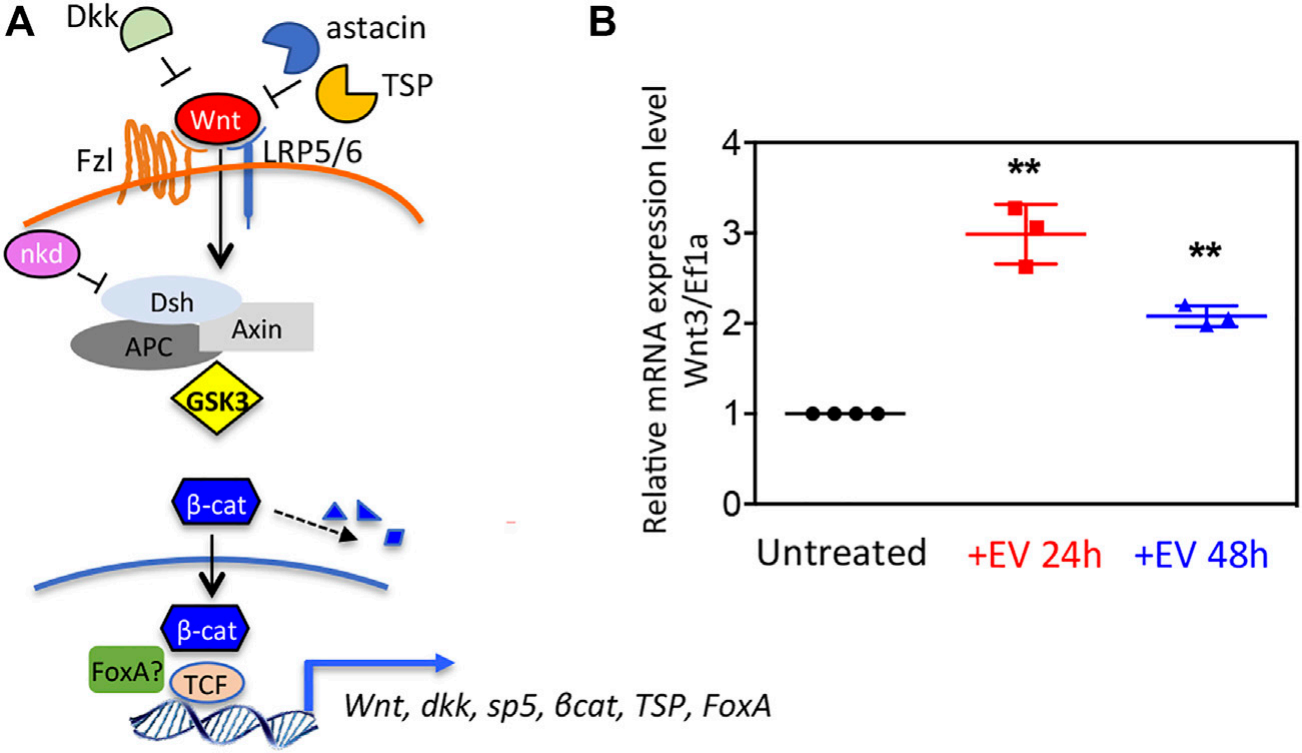
Wnt signaling activation via *Hydra* EV. **(A)** Schematic view of the canonical Wnt/β-catenin pathway at cellular level, in the activated state, showing extracellular and intracellular regulators identified in the *Hydra* EV transcriptome. **(B)** Relative mRNA expression levels of *Wnt3/Ef1*α. Whole polyps were treated for the indicated period with EVs freshly isolated from Hydra medium, then processed for RNA extraction and qRT-PCR. Data represent the average of three biological replicates (*n* = 60), each performed in triplicate and are presented as mean ±SD. Statistics was tested by two-sided unpaired t test. **p < 0.005 (*p* = 0.0015 for 24 h analysis and *p* = 0.0037 for 48 h analysis).

The finding in the EV transcriptome of both positive and negative regulators of the Wnt/β-catenin pathway (Figure 3A) prompted us to evaluate the EV bioactivity in receiving polyps. *Wnt3* gene transcript levels were shown upregulated in polyps treated with EVs, both at 24 and 48 h post treatment (Figure 3B), suggesting for the first time the involvement of *Hydra* EVs in the exogenous activation of Wnt signalling.

**FIGURE 4:**
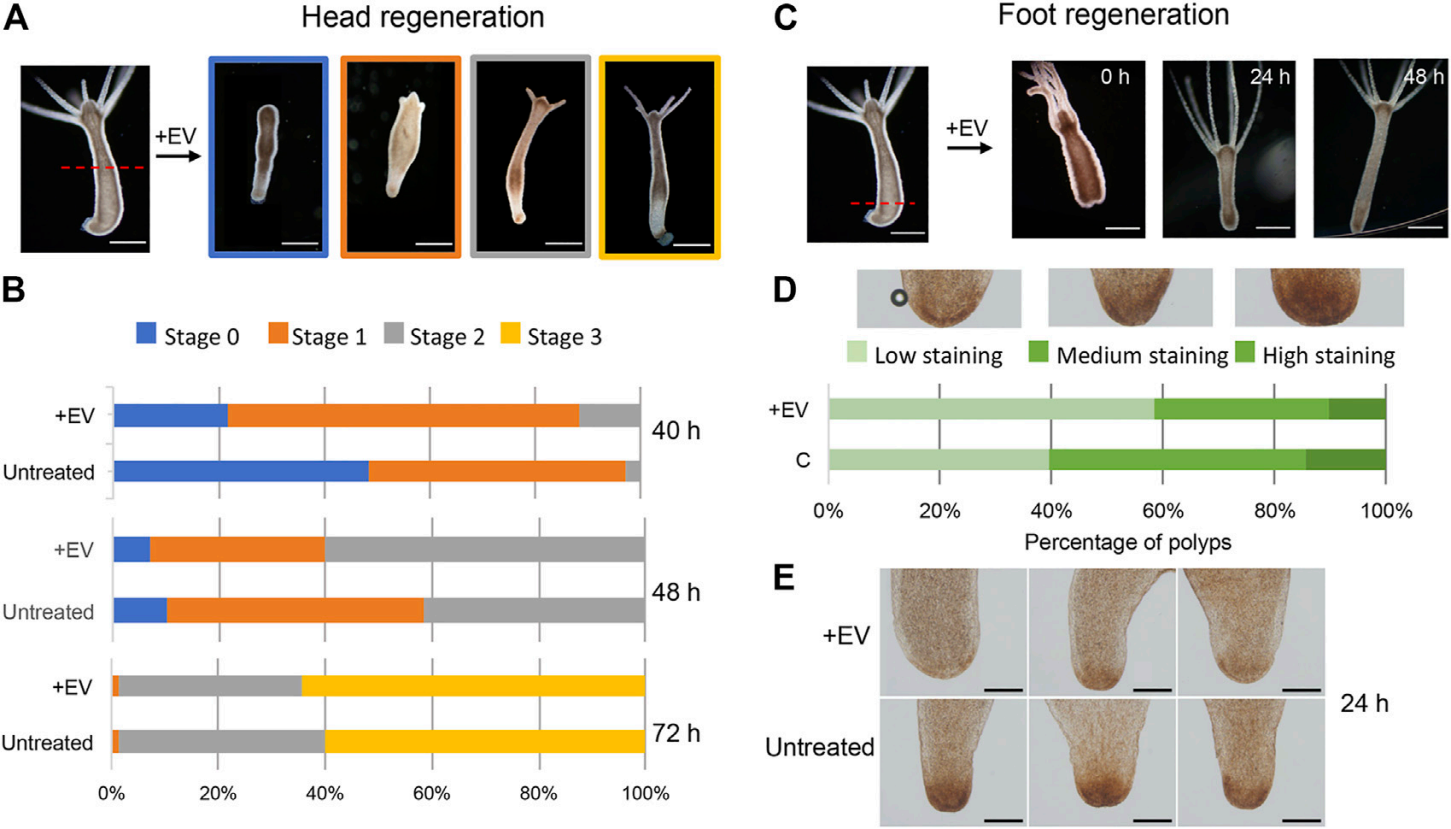
*Hydra* head and foot regeneration are modulated by EVs. **(A)** Light microscopy images of regenerating polyps, grouped in four categories according to progressive developmental stage. **(B)** Histograms report the distribution of developmental stages at 40, 48 and 72 h post midgastric amputation (shown by the red dotted line). The bar color corresponds to the regenerative stages shown in **(A)**. Data represent the average of three independent experiments (*n* = 70). Differences in the distributions of untreated and treated polyps were statistically evaluated using Chi squared test. At 40 h χ^2^ = 39.353 with 2 degrees of freedom. Two-tailed ***p value = 0.000057; at 48 h χ^2^ = 9.958 with 2 degrees of freedom. Two-tailed *p value = 0.0069; not significant differences resulted at 72 h: χ^2^ 0.548 with 2 degrees of freedom. The two-tailed *p* value = 0.7605 **(C)** Light microscopy images of foot regenerates at different time post amputation (red dotted line). **(D)** Distribution of foot regenerating polyps grouped in three categories: light, medium and dark green, corresponding to low, medium and high peroxidase staining, determined as DAB positive area by using the ImageJ software. Experiments were performed in triplicate (*n* = 45). χ^2^ = 7.062 with 2 degrees of freedom. two-tailed *p value: 0.0293. **(E)** Representative images of foot regenerating polyps (untreated and treated with EVs) 24 h p.a.

Next, the bioactivity of the EV molecular cargo was evaluated in the context of the regeneration process. Freshly prepared EVs were added to polyps amputated at midgastric level (50% of body column length), and the head regeneration efficiency was evaluated by morphometric analysis. Stumps of progressive developmental stage (stages 0, 1, 2 and 3) were monitored at regular intervals, starting at 24 h post amputation (p.a.) (Figure 4A). At each time point significant differences were observed in the distribution of developmental stages, with the EV-treated stumps always presenting more advanced stages compared to the controls (Figure 4B). Similar results were also obtained when the amputation was sub-hypostomal (80% of upper body length) (Supplementary Figure S6). These data indicate a clear enhancement effect played by EVs in the regeneration of the head, suggesting that the EV cargo may act as an exogenous source of molecular signals. The impact of EVs on the ability of the lower body cells to regenerate a new foot was evaluated by bisecting whole polyps at 20% of the body length (Figure 4C). The absence of distinctive morphological changes that characterize this process led us to use biochemical assays to monitor the differentiation of foot specific cells, based on the detection of peroxidase activity. This enzymatic activity is used as specific marker of basal disk cell differentiation, producing a strong signal at 36–48 h p.a. (Hoffmeister and Schaller, 1985). While control regenerates showed a clear peroxidase-positive basal disk at 36 h p.a., there was only a faint staining in the basal disk region at this time, indicating an inhibitory effect played by EVs on foot regeneration process. The observed modulation of the regeneration confirms the presence of important messages into the EVs, as detected by the transcriptome analysis, and suggest a possible regulatory role in *Hydra* biology.

## Discussion

The production and release of EVs has been documented in all three domains of life, both in unicellular and multicellular organisms. Functional studies have demonstrated their involvement in a large number of processes, including morphogenesis and development, while increasing evidences suggest that they may also participate in intra- and inter-species communication (Buck et al., 2014; Cruz et al., 2018; Cai et al., 2019). The evolutionary conservation of this form of communication poses important questions about the role it might have had on the generation of complex life as we know it today (Woith et al., 2019; Gill et al., 2019). Apart from our work detecting EVs on *Hydra* ectodermal cell surface (Marchesano et al., 2013), so far no evidences documented their presence in cnidarians, where they might act as signals for cell-cell communication, or as long range signals across species, or even kingdoms of aquatic life. We purified and characterized EVs from *Hydra* culture medium by differential centrifugation, characterized their round-shaped and double-layered morphology via TEM and estimated the size in the range consistent with exosome EV subtype (Doyle and Wang, 2019). Proteomic analysis revealed numerous exosome markers, often related to their biogenesis route and cargo sorting mechanisms (Jeppesen et al., 2019; Juan and Furthauer, 2018). This evidence, together with the morphology, size and mRNA loading strongly suggests that the EVs isolated are highly enriched in this vesicle subtype. Most of the identified proteins correlate with the transcripts, including proteinases, catalytic proteins and transcription factors, suggesting the delivery of key molecular messages for early and late responses in the receiving cells. Stress induced molecular chaperons were found at the protein level (i.e., Hsp70, Hsp71, Supplementary Table S1) and among the most abundant transcripts of endodermal- and interstitial cell-specific transcripts (*hsp90* and *hsp70* respectively (Supplementary Table S4). Their presence, together with other stress related transcripts (i.e., stress induced phosphoprotein), superoxide dismutase and thioredoxin protein (disulphide isomerase) reveals an important role played by EVs in the stress response, conveying long distance messages to support survival and adaptation as described in literature. Similarly, detection of proteins and transcripts of the serine protease inhibitor kazal (Chera et al., 2009a) suggests a possible involvement of EVs in the starvation response, by preventing excessive autophagy. Indeed, induction of autophagy during starvation is the main survival strategy in *Hydra* and kazal was shown to support cell survival by preventing excessive autophagy (Chera et al., 2006). As EV harvesting occurs under starvation, kazal may support the animal survival to this condition, mirroring natural defence mechanisms acting through EVs.

In addition to the above-mentioned factors, a multitude of signalling components belonging to BMP, Notch and Wnt/βcatenin pathways, were identified among mRNA transcripts. Our focus on the Wnt/β-catenin signalling pathway, searching for positive and negative regulators of it in EVs, is due to the major role played by this pathway in body axial patterning. In *Hydra* two stable tissue organizers located at the oral and aboral poles are responsible for the tissue polarity (Bode, 2009). While the molecular bases of foot organizer are still largely unknown, the head organizer relies on the canonical Wnt/βcatenin pathway and other integrating signalling pathways, such as the Notch-Delta (Broun et al., 2005; Gee et al., 2010; Munder et al., 2013). Using classical tissue manipulation techniques (Macwilliams, 1983) and more recently gene transcript profiling (Chera et al., 2009b; Wenger et al., 2019; Cazet et al., 2021) the presence of long-range inhibitory signals in *Hydra* head organizer has been supposed, acting in adults polyps to maintain the oral-aboral polarity and preventing the formation of ectopic organizers elsewhere in the body. Amputated organizers may transiently increase the ability of wounded tissue throughout the body column to form secondary axes, while during foot regeneration these inhibitory signals would prevent ectopic head formation at aboral-facing amputations (Cazet et al., 2021). To date, these signals have not been identified and in the present work we foresee the possibility that they may rely on EVs released in the extracellular medium. We demonstrated that treating amputated polyps with EVs at an empirical dosage, likely much higher when compared to the physiological EV production, impacts on *Hydra* regeneration, and this effect may be due to the peculiar molecular cargo of EV collected under these conditions. While under physiological condition EVs may contribute to tissue organizer activity (Macwilliams, 1983; Cazet et al., 2021) by enhancing the head regeneration at the oral facing amputation region, and preventing the formation of multiple heads at the opposite end, under our condition EVs may inhibit foot regeneration, as a result of an unique blend of molecular signals. Indeed in addition to mRNA, tRNA and lncRNA here detected, EVs are known to vehicle small RNA, lipids, DNA and other metabolites (Veziroglu and Mias, 2020), opening the path to further analyses for a deeper characterization of *Hydra* EVs and unravel their role in developmental signalling and many other aspects of *Hydra* biology.

The alignment of EV transcriptome with the scRNA-seq atlas recently produced (Siebert et al., 2019) allowed to extract important information not only on the molecular composition of the EV cargo but also on the cell sources of EV transcripts. Under our condition of EV purification multiple cell types appeared involved in the loading and release of EVs. In addition to ectodermal cells expected to release EVs, as facing the external medium, a large contribute to the EV transcriptome matched transcripts specific of the interstitial stem cell lineage, dispersed throughout the body column, especially neurons. This suggests that EVs may mediate the information flow within the *Hydra* non overlapping neural networks (Dupre and Yuste, 2017). Interestingly, the sub-clustering analysis (Supplementary Figure S5) reveals that within the interstitial cell lineage a large fraction (18.7%) of the transcripts are germ line specific, suggesting the possibility that under the mild stressing condition used for EV collection the cells may release signals to induce gametogenesis and guarantee species survival. Finally, a minor fraction of transcripts was specific of endodermal cells, especially gland cells, which is consistent with their location, facing the gastric cavity, and their molecular repertoire of enzymatic activities and signalling factors, such as those restricting the Wnt signal in the hypostome (Guder et al., 2006; Lommel et al., 2018; Ziegler et al., 2021). A comparative analysis between EVs produced by transgenic lines tagged in each cell lineage would help in the future to confirm this *in silico* evidence.

Concerning the possible role *in vivo*, the EV information toolbox may be used for cell-cell communication to maintain homeostatic condition, i.e., axis polarity, regulate animal size and tissue growth. In other conditions, i.e., during budding, regeneration or stress response, they may act to orchestrate coordinated cell responses between distant tissue regions, or among different polyps. In this work we purified EVs from the medium of healthy polyps mirroring the condition used to enhance EV production from cell culture, i.e. starvation and crowding (Théry et al., 2006) and our omics and functional analyses are the outcomes of this particular state. EV may vary their molecular cargo under certain conditions, such as stress, reproduction, temperature changes, adapting the molecular information to deliver diverse messages to the cells, from hydrophobic signals to lipids, second messengers, or nucleic acids. We believe that by comparing the EV content produced under different physiological states different outputs might be produced, leading to decipher the “code” used by cells to communicate to distant cells or organisms and to coordinate precise responses to environmental challenges, which is of outmost importance in aquatic environments.

## Data Availability

The mass spectrometry proteomics data have been deposited to the ProteomeXchange Consortium via the PRIDE (Perez-Riverol et al., 2019) partner repository with the dataset identifier PXD027774. RNA-seq data were deposited in NCBI’s Gene Expression Omnibus (GEO) and are accessible through GEO Series accession number GSE180886.
