# Supplementary material for: The Aquatic Invertebrate Hydra vulgaris Releases Molecular Messages Through Extracellular Vesicles
Source: Front Cell Dev Biol. 2021 Dec 20;9:788117. doi: 10.3389/fcell.2021.788117 (PMC8721104; doi:10.3389/fcell.2021.788117)
Supplement: Supplementary file 1 [file Presentation1.pdf]

# Supplementary Materials

The aquatic invertebrate *Hydra vulgaris* releases molecular messages through extracellular vesicles

Maria Moros[1,2#,] Eugenio Fergola[2#], Valentina Marchesano[2], Margherita Mutarelli[2], Giuseppina Tommasini[2], Beata Miedziak[2,$], Giuliana Palumbo[2], Alfredo Ambrosone[2,&], Angela Tino[2], Claudia Tortiglione[2*]

1 Instituto de Nanociencia y Materiales de Aragón(INMA), Zaragoza, Spain

2 Istituto di Scienze Applicate e Sistemi Intelligenti "E.Caianiello", Pozzuoli, Italy

Present address

& Department of Pharmacy, Biomedical division, University of Salerno, Italy

$ University of Rzeszów, Faculty of Biotechnology, Rzeszów, Poland

* Corresponding author: claudia.tortiglione@cnr.it

Supplementary information includes:

Fig. S1. Morphological characterization of Hydra EV
Fig. S2. in vivo assembly of AuNP on Hydra EV
Fig. S3. Uptake of Hydra EV into Hydra tissue
Fig. S4. EV protein identification by MALDI-TOF/TOF
Fig. S5. Subclustering EV transcripts into single cell type
Fig. S6. Influence of EV on Hydra head regeneration
Table S1. List of 52 proteins identified in EV by LC-ESI-MS/MS analysis
Table S2 RT-PCR validation using presence/absence analysis
Table S3. Primer sequences used for RT-PCR analysis
Table S4. List of EV transcripts subclustered according to unique cell type expression
Table S5. List of top ten EV transcripts matching the scRNA-seq atlas

**Supplementary file 1**. Full list of transcripts detected in the alignment against the NCBI Hydra vulgaris reference transcriptome (assembly Hydra_RP_1.0)
**Supplementary file 2**. Full list of transcripts detected in the alignment against the single cell RNAseq reference transcriptome (assembly GHHG01.1)

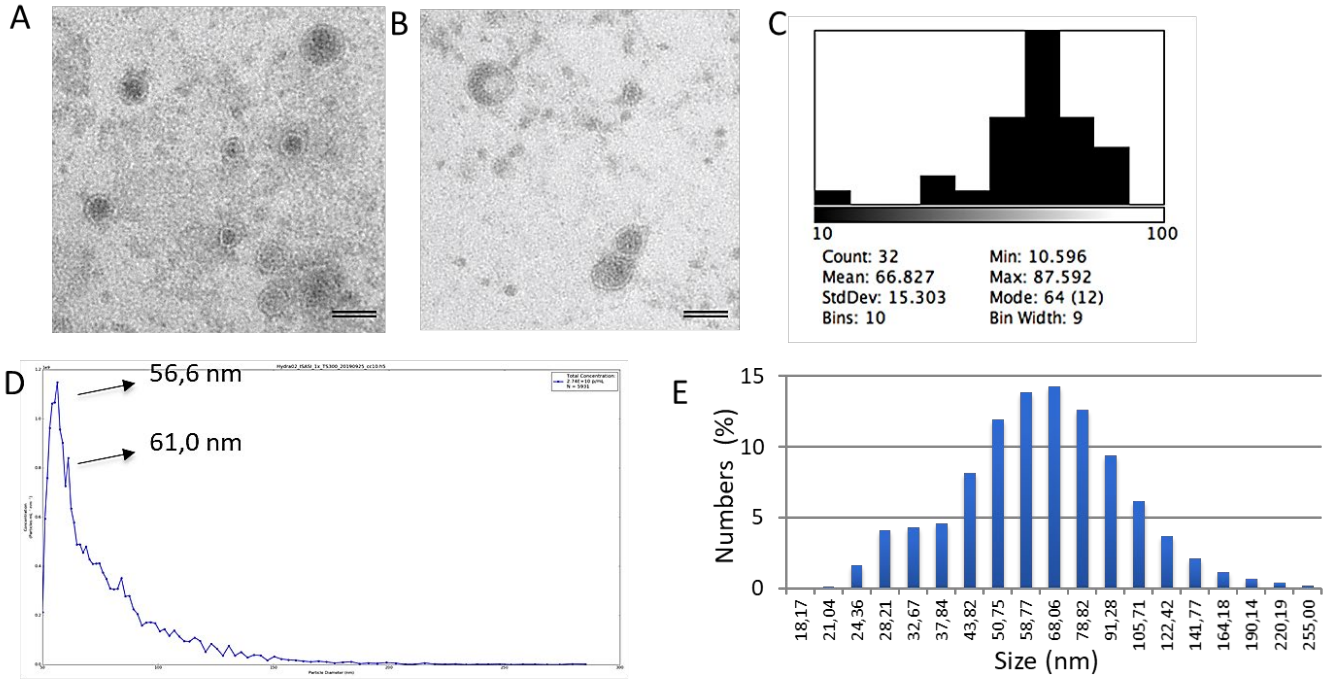


**Fig. S1. Morphological characterization of Hydra EV.**

A-B) TEM images show the double membrane limiting electron dense core of the EV. Scale bars 50 nm. C) Size distribution of vesicles identified by TEM D) MRPS measurements showing the distribution of the EV size, presenting two major peaks at 56,6 and 61 nm (using a TS-300 cartridge). These measurements well correlated with the values obtained using the TS-400 cartridge, showed in Figure 1D. E) Dynamic light scattering analysis shows an average size of Hydra EV peaking at 68 nm.

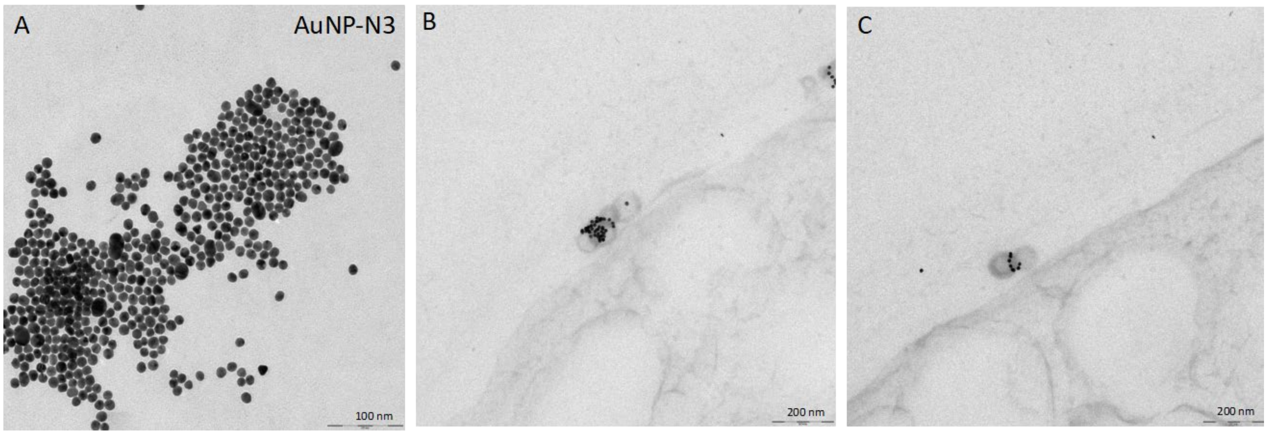


**Fig. S2*. In vivo* assembly of AuNP on Hydra EV**

A) TEM image (TEM, T20 200KeV; FEI) of positively charged AuNPs used in this study shows the high monodispersity of the gold core of AuNP-N3 of an average size of 14 nm. B-C) Polyps were incubated with 25 nM AuNP, then extensively washed and processed 48 h later for TEM analysis. AuNPs appear attached to nanosized vesicles outside the cell membrane, within the glycocalyx. Scale bars, 100 nm in A, and 200 nm in B, C.

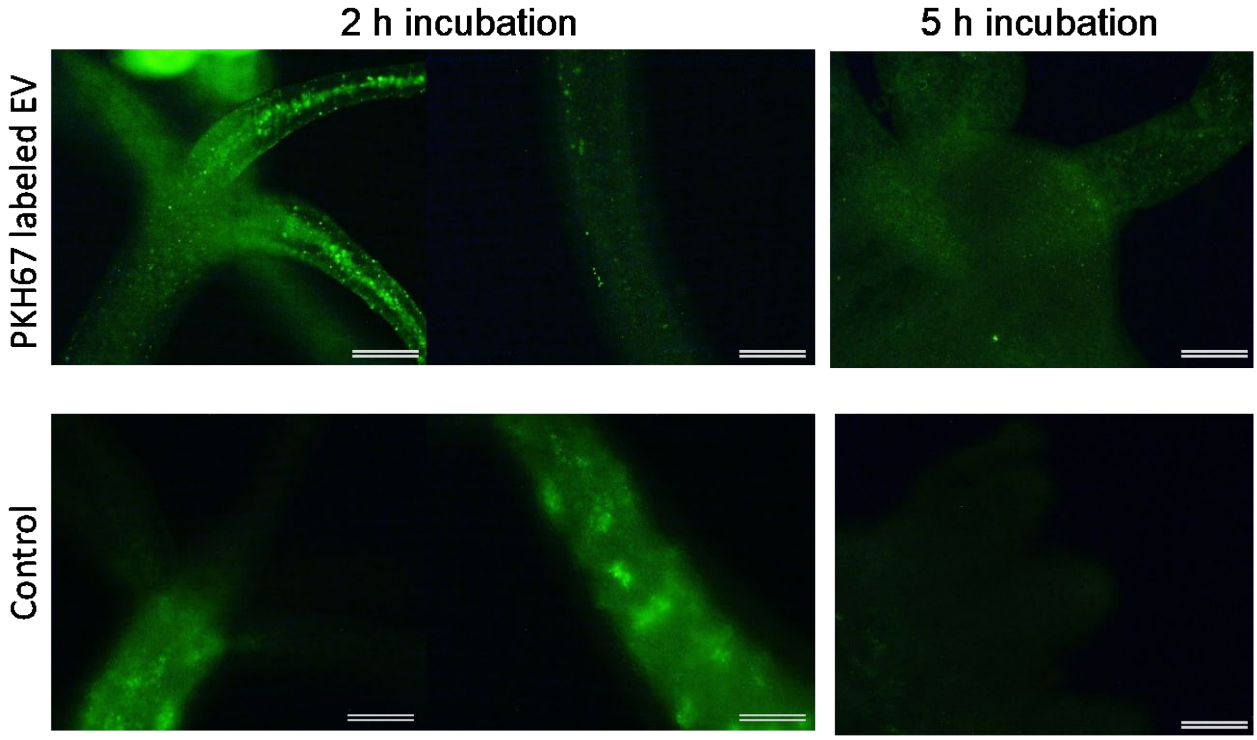


**Fig. S3. Uptake of Hydra EV into Hydra tissue**

Living Hydra were treated with freshly EVs labelled with PKH67 derived from a standard EV preparation (250 polyp into 10 ml of medium). Upper panel: 2 h post incubation, labelled EVs are internalized in the animal tissues as shown in the upper part of the figure where a green, fluorescent granular signal is detectable both in the tentacles (left) and the body column (right). After 5 h of incubation, this signal was hardly detectable, possibly indicating the processing of EVs inside the cells. Lower panel: polyps treated with a negative control solution (PKH67 in Hydra medium) show a diffuse green fluorescent pattern due to the free dye internalization. After 5 h this signal almost disappears. Scale bars, 200 µm.

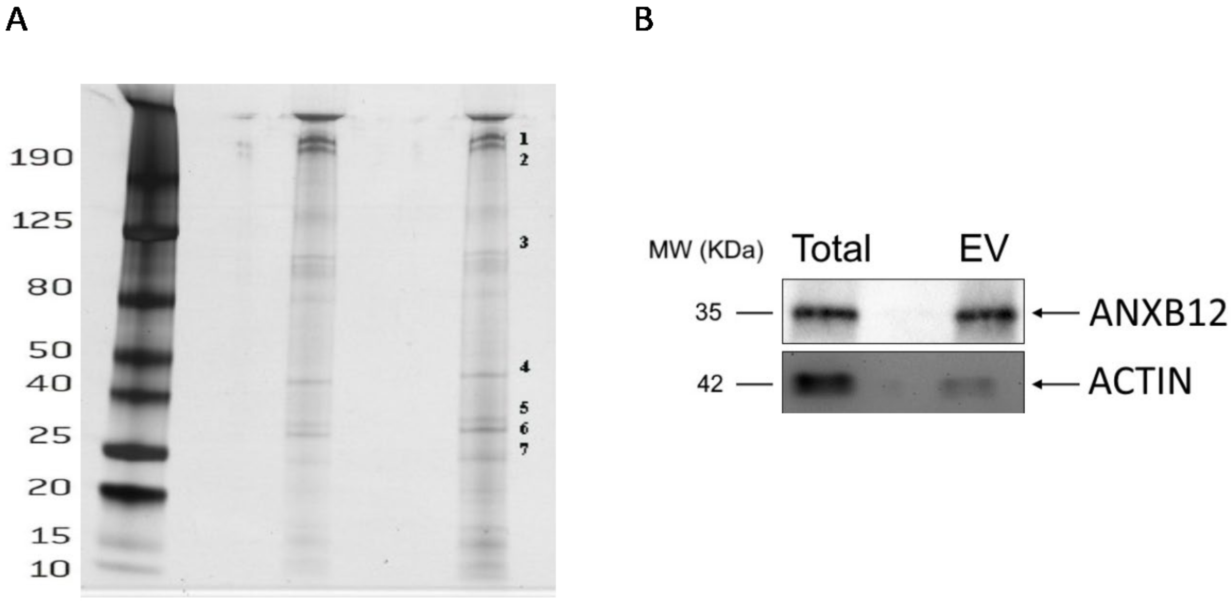


**Fig S4**. **EV protein identification by MALDI-TOF/TOF**

A) Proteins extracted from EVs were run in a 7.5% SDS-PAGE gel. After silver staining, 7 bands were excised from the gel and processed for MALDI-TOF/TOF analysis. Actin was identified from band n.4, Annexin B12 from band 5. 2 biological replicas were performed. B) Western blot analysis in whole-body protein extracts (Total) and EV fractions (EV) shows specific cross reactions to anti Hydra Annexin B12, and anti β actin antibodies

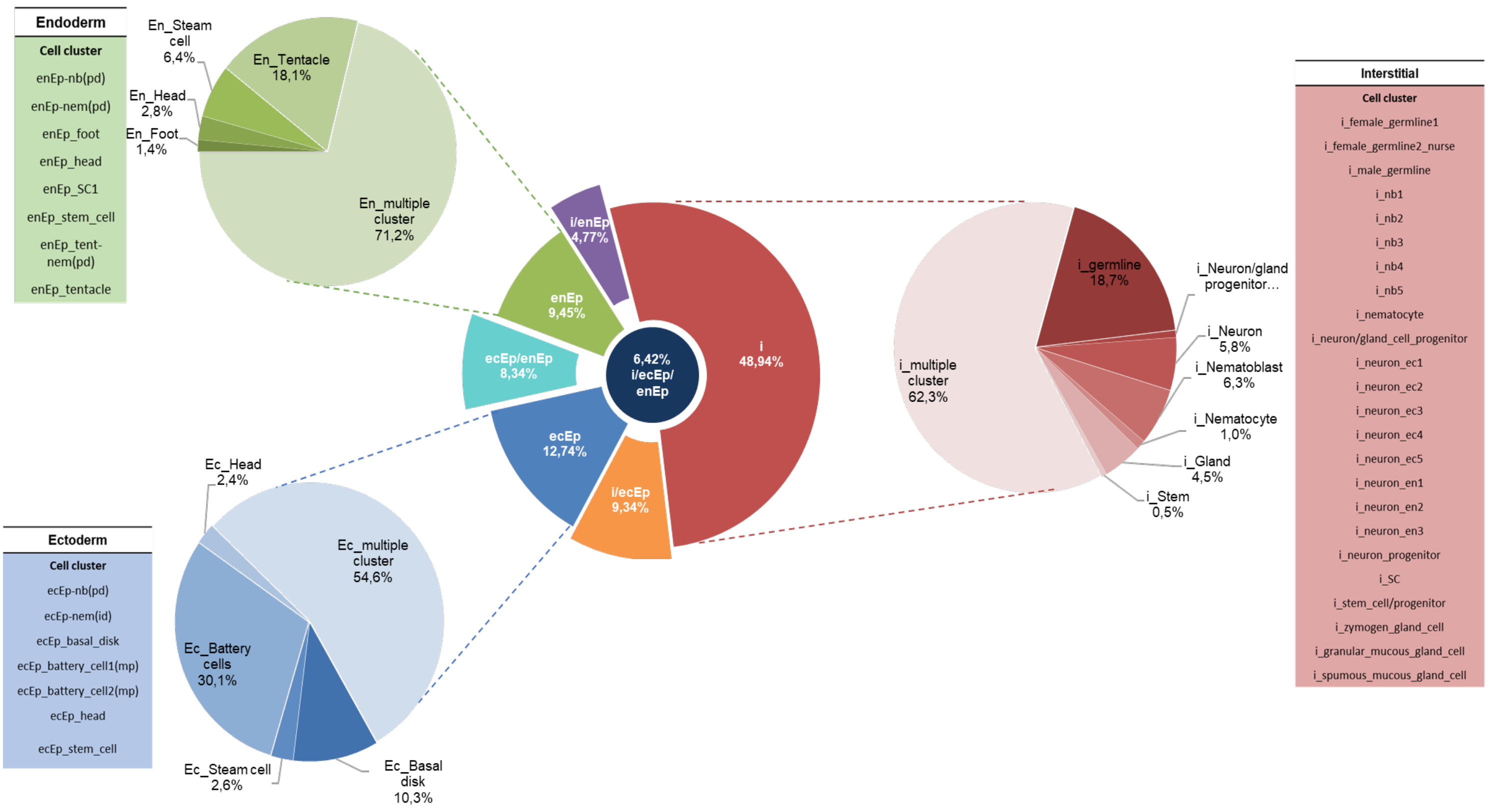


**Fig.S5. Subclustering EV transcripts into single cell types**

Hydra EV-associated genes were clustered according to ectodermal, endodermal and interstitial cell lineages using a recently-published scRNA-seq atlas (Siebert, Farrell et al. 2019) and expressed as percentages. EV transcripts common to the three lineages are shown in dark blue, while those unique of the interstitial cell lineage (in red) represent the major portion. The amount of ectodermal and endodermal transcripts (in blue and green, respectively) are similar, to those shared between two lineages (interstitial and ectodermal, in orange; ectodermal and endodermal, in azure; interstitial and endodermal in violet).

For each cell lineage EV transcripts matching multiple cell cluster represent the wider portion, while a minority match with single cell clusters, listed beside each distribution.

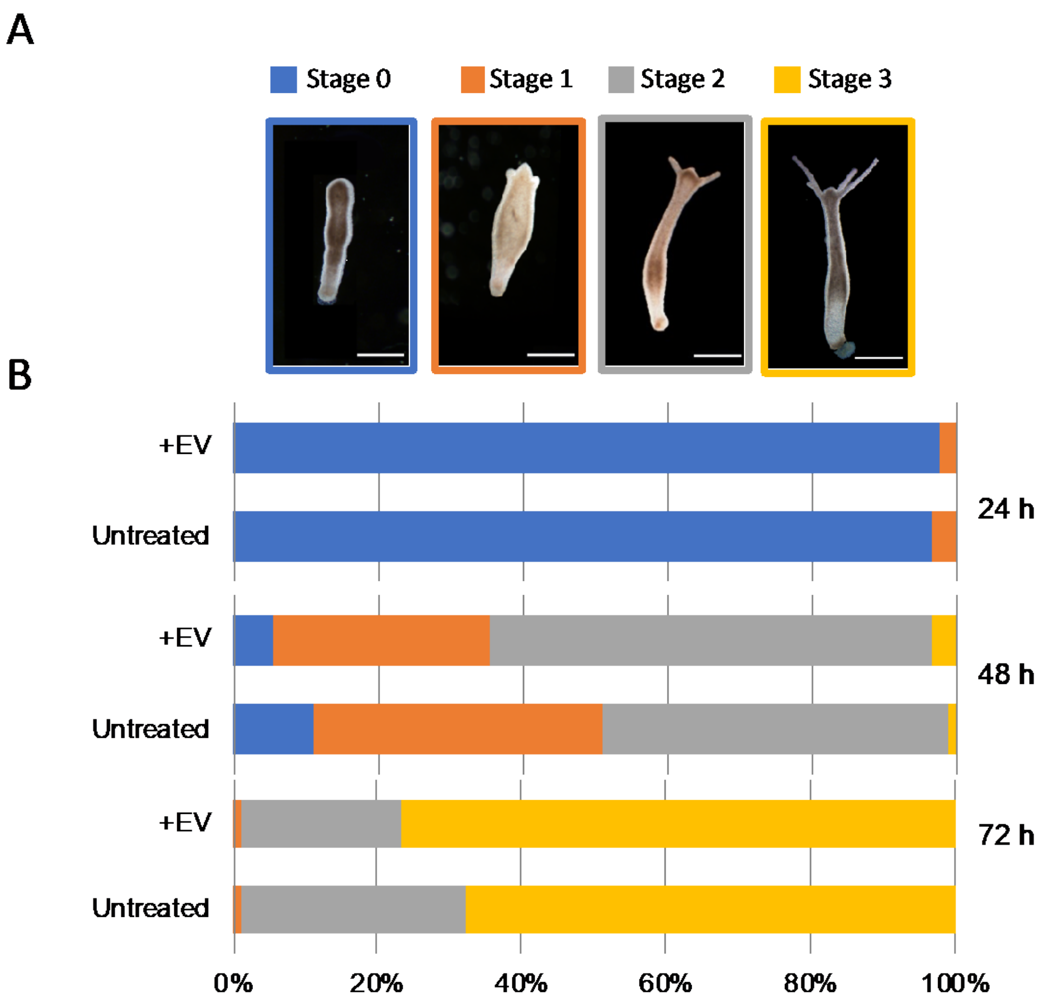


**Fig. S6. Influence of EV on Hydra head regeneration.**

Groups of 30 animals were bisected at subhypostomal level and allowed to regenerate in presence of EV, or in normal medium (untreated). A) At 24 h, 48 h and 72 h p.a. animals were inspected for viability and regeneration stage: stage 0 indicates wound closure; stage 1 indicates the presence of tentacle buds; stage 2 indicates new emerging tentacles, stage 3 indicates growth tentacles. B) Histograms report the distribution of developmental stages at 24 h, 48 h and 72 h p.a. The bar color corresponds to the regenerative stages shown in A. Animals treated with EV present more advanced stages than untreated animals. Data are means of three independent experiments (n=90). Statistical analysis performed at 48 h p.a. shows Chi squared =12.099 with 3 degrees of freedom. The two-tailed **P value= 0.0071. At 72 h Chi squared equals 3.335 with 2 degrees of freedom. The two-tailed P value equals 0.1887 (not significant).

| Accession No. | Protein Name | Putative Function | Matched Peaks | Matche Peptide | Mascot Score | Exocarta | Conserved Domain | Pfam Codes InterPro Code |
|---|---|---|---|---|---|---|---|---|
| CDG70628 | Actin, cytoplasmic 1, partial | Cytoskeleton component | 18 | 16 | 235 | Yes | Actin | pf0002 |
| XP_002161913 | Tubulin beta chain | Cytoskeleton component | 10 | 8 | 169 | Yes | Tubulin | pf0009 |
| XP_002156816 | Uncharacteriz ed protein LOC10020711 8 | Metalloproteinas e with Thrombospondin motifs | 5 | 5 | 130 | --- | Thrombospon din type 1 repeats | IPR000884 |
| XP_012558692 | Fibrillin-1 | Microfibril assembly | 11 | 10 | 125 | Yes | Calcium-binding EGF domain | pfam07645 |
| XP_002166874 | Fibrillin-2 isoform X1 | Microfibril assembly | 9 | 7 | 107 | Yes | EGF domain | pfam12947 |
| NP_001296699 | Annexin B12 | Calcium-dependent membrane binding | 7 | 4 | 100 | No | Annexin | pfam00191 |
| XP_002159321 | Syntenin-1-like isoform X2 | Biogenesis of exosomes | 9 | 8 | 99 | Yes | PDZ domain | IPR001478 |
| NP_001296663 | Elongation factor EF1-alpha | Translation elongation factor | 11 | 10 | 89 | Yes | Elongation factor Tu GTP binding domain | pf00009 |
| XP_004212135 | Protocadherin Fat 4, partial | Cell-cell adhesion | 7 | 7 | 89 | Yes | | |
| XP_002159997 | Uncharacterized protein LOC100198870 | ------- | 5 | 4 | 86 | ------- | ------- | ------- |
| XP_012559193 | Uncharacteriz ed protein LOC10020809 3 | -------- | 9 | 7 | 86 | ------- | ------ | |
| XP_004205459 | Histone H4 | Nucleosome assembly | 8 | 7 | 83 | Yes | CENP-T Domain | pf15511 |
| XP_002154154 | Latent-transforming growth factor beta-binding protein 4-like | Extracellular matrix constituent | 12 | 12 | 80 | Yes | Calcium-binding EGF domain | pfam07645 |
| XP_012556685 | Zinc finger MYM-type protein 1 | Transcriptional repressor | 9 | 9 | 79 | Yes | EF-hand domain | IPR002048 |
| CDG71416 | Heat shock cognate 71 kDa protein | Molecular chaperon | 8 | 8 | 73 | Yes | hsp70 protein domain | pf00012 |
| ABC25030 | Heat shock protein 70 | Stress-induced molecular chaperon | 9 | 9 | 73 | Yes | hsp70 protein domain | pf00012 |
| XP_004212674 | Protocadherin Fat 1, partial | Cell-cell adhesion | 17 | 17 | 71 | Yes | Cadherin-like domain | IPR002126 |
| XP_012558915 | Uncharacteriz ed protein LOC10019713 0 | Chitin binding | 2 | 2 | 71 | --- | Chitin binding Peritrophin-A domain | pfam01607 |
| XP_012554603 | Uncharacteriz ed protein LOC10020828 5 | CUB extracellular domain | 11 | 11 | 69 | --- | EGF-like domain | IPR000742 |
| XP_002166847 | Uncharacteriz ed protein LOC10019842 7, partial | ------------------------- | 2 | 2 | 67 | ---- | ------ | ------ |

| | | | | | | | | | |
|---|---|---|---|---|---|---|---|---|---|
| XP_004209567 | Histone H1-delta | Nucleosome assembly | 5 | 5 | 63 | Yes | linker histone H1 and H5 family | pfam00538 |
| XP_012553809 | Annexin A4-like | Calcium-dependent membrane binding | 14 | 13 | 61 | Yes | Annexin | pfam00191 |
| AAW82079 | Thypedin | Foot formation stimulator | 5 | 5 | 57 | No | Thymosin | pf01290 |
| XP_002163070 | Ubiquitin-40S ribosomal protein S27a | Vesicle cargo sorting | 3 | 2 | 57 | No | Ribosomal protein S27a Ubiquitin-like (Ubl) domain | pfam01599 cd01803 |
| XP_002165348 | CD151 antigen | Exosome marker | 5 | 5 | 56 | Yes | Tetraspanin family | pfam00335 |
| AAN87350 | 14-3-3 protein B | Growth adaptation to food supply | 2 | 2 | 54 | No | 14-3-3 protein domain | pfam00244 |
| CAJ33888 | Putative serine protease inhibitor | Protective function against excessive autophagy | 5 | 5 | 54 | No | Kazal type serine protease inhibitors | Pfam 00050 |
| XP_002156827 | Ras-like GTP-binding protein RHO | Signal transduction | 2 | 2 | 52 | Yes | RhoA-like | IPR005225 |
| XP_012562569 | Uncharacterized protein LOC100200012 | Cell adhesion | 5 | 5 | 51 | --- | vWFA/TSP_1 | Pfam00090 |
| XP_004211099 | Usherin-like, partial | Integral component of membrane | 2 | 2 | 47 | No | Laminin N-terminal (Domain VI) | PF00055 |
| CDG70601 | 40S ribosomal protein S3a, partial | Ribosome assembly | 6 | 6 | 46 | Yes | Ribosomal S3Ae family | pfam01015 |
| XP_012564127 | Uncharacterized protein LOC101234629 | Calcium-binding protein | 9 | 7 | 44 | --- | Calcium-binding EGF domain | PF07645 |
| XP_012565905 | Uncharacterized protein LOC105849991, partial | ------ | 4 | 3 | 44 | --- | ------ | ------ |
| CDG71497 | ATP synthase subunit beta, mitochondrial, partial | Cellular respiration | 10 | 10 | 44 | Yes | ATP synthase $\alpha/\beta$ family, nucleotide-binding domain | PF00006 |
| XP_002162060 | Programmed cell death 6-interacting protein | Multivesicular body biogenesis | 10 | 9 | 43 | Yes | BRO1-like domain | PF03097 |
| XP_002155023 | Polyubiquitin-B | Vesicle cargo sorting | 11 | 7 | 43 | Yes | Ubiquitin family | pf00240 |
| XP_012566106 | Uncharacterized protein LOC100203042, partial | Thrombospondin type 1 | 3 | 2 | 42 | --- | Spondin-like TSP1 domain | pfam19028 |
| XP_012554672 | cAMP-dependent protein kinase type II regulatory subunit-like isoform X2 | cAMP-mediated signalling pathway | 5 | 4 | 42 | Yes | Cyclic nucleotide-binding domain | IPR000595 |
| XP_004208264 | Uncharacterized serine-rich | ------- | 2 | 2 | 41 | ----- | ------ | ------ |

| | | | | | | | | |
|---|---|---|---|---|---|---|---|---|
| | protein C215.13-like | | | | | | | |
| XP_012564784 | Von Willebrand factor D and EGF domain-containing protein-like, partial | Cell surface receptor signaling pathway | 6 | 6 | 41 | No | Calcium-binding EGF domain | pfam07645 |
| XP_004208788 | Tubulin alpha-1D chain | Cytoskeleton component | 5 | 4 | 41 | Yes | Tubulin C-terminal domain | PF03953 |
| XP_002158871 | Flagellar calcium-binding protein-like | Calcium ion binding | 4 | 3 | 40 | No | EFh superfamily | Pfam13833 |
| CDG71487 | Protein disulfide-isomerase | Disulfide isomerase | 5 | 4 | 40 | Yes | Thioredoxin domain | IPR013766 |
| XP_012553917 | Malate dehydrogenase, cytoplasmic-like | Krebs cycle | 3 | 3 | 38 | Yes | Lactate/malate dehydrogenase, NAD binding domain | PF00056 |
| AAQ13347 | Ribosomal protein L10 | Ribosome pathway | 3 | 2 | 36 | Yes | Ribosomal protein L16p/L10e | PF00252 |
| XP_004209147 | Cell division control protein 42 homolog | small GTPase mediated signal transduction | 2 | 2 | 36 | No | Small GTP-binding protein domain | IPR005225 |
| XP_012557607 | Uncharacterized protein LOC100208299 | Chromosome segregation protein SMC | 6 | 6 | 34 | -- | SMC_prok_B | |
| XP_002162887 | ADP-ribosylation factor 1-like 2 | Retrograde transport, endosome to Golgi | 2 | 2 | 34 | Yes | Small GTP-binding protein domain | IPR005225 |
| AEW90237 | Voltage-gated sodium channel Nav2.1, partial | Neuronal signaling in Hydra | 15 | 15 | 34 | No | Ion transport protein | pfam00520 |
| XP_012560741 | Uncharacterized protein LOC105846485, partial | -------- | 7 | 6 | 32 | -- | YlqF_related_GTPase | ------ |
| XP_012562547 | Mitotic spindle assembly checkpoint protein MAD1-like | Mitotic spindle assembly checkpoint signaling | 11 | 11 | 32 | No | Mitotic checkpoint protein | PF05557 |
| XP_004207315 | Uncharacterized protein LOC101240603 | Reverse transcriptase | 3 | 2 | 32 | --- | RT-like | |

**Table S1**. List of 52 proteins identified in EV by LC-ESI-MS/MS analysis, mascot score >32, matched peptide >2

| TARGET GENE | $C_t$ MEAN |
|---|---|
| *Hsp70* | 24.86 ± 1.85 |
| *Hsp70* (NTC) | 37.60 ± 1.96 |
| | |
| *β-cat* | 27.07 ± 0.27 |
| *β-cat* (NTC) | 38.51 ± 2.11 |
| | |
| *Wnt3* | 30.22 ± 0.75 |
| *Wnt3* (NTC) | 38.19 ± 2.12 |
| | |
| *Actin* | 25.60 ± 0.5 |
| *Actin* (NTC) | 37.43 ± 2.05 |

**Table S2 RT-PCR validation using presence/absence analysis**

Ct mean ± SD were obtained from two independent biological experiments performed in triplicate.

NTC= no template control

| Gene | Accession No | Forward Primer | Reverse Primer |
|---|---|---|---|
| Wnt3 | NM_001287363 | 5’-CAACAAGGCTGGACGAGAGG-3’ | 5’-CTGAAATGGGGCTGTGAACG-3’ |
| *β*-catenin | U38624 | 5’-TGCACCTTCTTCACGCTCAATAG-3’ | 5’-GGCGCTCTTCACATTTTAGC-3' |
| Actin | XM_002154426 | 5’- AAGCTCTTCCCTCGAAGAATC-3’ | 5’- CCAAAATAGATCCTCCGATCC-3’ |
| Hsp70 | NM_001309695 | 5’-CGACGTATTCAGACAATCAAC-3’ | 5’-CAATTTGAGGAACACCTCTTG-3’ |
| Ef1*α* | NM_001309734.1 | 5’-CCAGGAGACAATGTCGGTTT-3’ | 5’-GCTTCAATGGCAGGATCATT-3’ |

**Table S3.** Primer sequences used for RT-PCR analysis

| Cell Cluster | Gene ID | Gene ID (NCBI) | Gene name | Homology (OrthoDB) |
|---|---|---|---|---|
| Ec_basal_disk | t11369aep | 100199560 | TCTP | TPT1 [Homo sapiens] NP_001273201.1 |
| | t16171aep | 105845407 | Uncharacterized | galaxin-like isoform X2 [Danio rerio] XP_005163007.1 |
| | t9810aep | 105847572 | Flocculation protein FLO11-like | IFT140 [Homo sapiens] XP_016879399.1 |
| | t11479aep | 100207237 | Uncharacterized | C-type lectin-like |
| | t13256aep | 105844822 | rhamnose-binding lectin-like | EVA1C [*Homo sapiens*] NP_478067.2 |
| Ec_battery_cell2(mp) | t11116aep | 100202036 | actin, non-muscle 6.2-like | POTEI [Homo sapiens] XP_016860221.1 |
| | t34888aep | 105846558 | lipopolysaccharide-induced tumor necrosis factor-alpha factor homolog | CDIP1 [Homo sapiens] NP_037531.2 |
| | t4993aep | 100215092 | inositol-tetrakisphosphate 1-kinase-like | ITPK1 [Homo sapiens] NP_055031.2 |
| | t30709aep | 105845211 | protein PFC0760c-like | / |
| | t35aep | 100202307 | ADP-ribosylation factor 6 | ARL4C [Homo sapiens] NP_001269360.1 |
| Ec_head | t24379aep | / | / | / |
| | t4117aep | / | / | / |
| | t33641aep | 100213910 | ATP synthase F(0) complex subunit B1, mitochondrial-like | ATP5F1 [Homo sapiens] NP_001679.2 |
| | t19284aep | 100209701 | ATP synthase subunit gamma, mitochondrial-like | ATP5F1C [Homo sapiens] NP_001001973.1 |
| | t16848aep | 100207790 | mitochondrial-processing peptidase subunit beta-like | UQCRC1 [Homo sapiens] NP_003356.2 |
| Ec_stem_cell | t19380aep | / | / | / |
| | t22090aep | / | putative leucine-rich repeat-containing protein DDB_G0290503 | / |
| | t26296aep | 100209179 | cold shock domain-containing protein E1-like | CSDE1 [Homo sapiens] NP_001229820.1 |
| | t13120aep | 100204386 | mitochondrial import receptor subunit TOM70-like | TOMM70 [Homo sapiens] NP_055635.3 |
| | t7502aep | 100211597 | prostaglandin E synthase 2-like | PTGES2 [Homo sapiens] NP_079348.1 |
| EnEp-nem(pd) | t38379aep | 100200982 | electron transfer flavoprotein subunit beta-like | ETFB [Homo sapiens] NP_001014763.1 |
| En_foot | t27064aep | 101240735 | Uncharacterized | POGK [Homo sapiens] XP_016857403.1 |
| | t31094aep | 100215335 | forkhead box protein D2-like | fkh-10 [Caenorhabditis elegans] NP_492676.2 |
| | t9657aep | 101238486 | neuropeptide Y receptor-like | CG13995 [Drosophila melanogaster] NP_608986.1 |
| | t10383aep | 100215397 | glycylpeptide N-tetradecanoyltransferase 2-like | NMT1 [Homo sapiens] NP_066565.1 |
| En_head | t16838aep | | | |
| | t20529aep | 100199243 | protein RER1-like | RER1 [Homo sapiens] NP_008964.3 |
| | t16864aep | / | Cytosolic 10-formyltetrahydrofolate dehydrogenase [Exaiptasia pallida] | / |
| | t1100aep | 101236386 | ras-specific guanine nucleotide-releasing factor RalGPS2-like | RALGPS2 [Homo sapiens] NP_689876.3 |
| | t34763aep | 100205238 | Uncharacterized | protein naked cuticle homolog 2-like [Propithecus coquereli] XP_012520768.1 |
| En_SC1 | t20542aep | 101235897 | hsp90aa1 | HSP90AA1 [Homo sapiens] NP_001017963.2 |
| En_stem_cell | t1207aep | | galaxin-2-like [Lingula anatina] | / |

| | | | | |
|---|---|---|---|---|
| | t6483aep | 100204864 | mitochondrial import inner membrane translocase subunit Tim17-A-like | TIMM17B [Homo sapiens] NP_001161419.1 |
| | t11367aep | 100211497 | cytochrome c oxidase subunit 4 isoform 2, mitochondrial-like | COX4I1 [Homo sapiens] XP_024305924.1 |
| En_SC1 | t22130aep | 100203295 | stress-induced-phosphoprotein 1-like | STIP1 [Homo sapiens] NP_001269581.1 |
| enEp_tent-nem(pd) | t33887aep | / | / | / |
| enEp_tentacle | t29036aep | / | / | / |
| enEp_tent-nem(pd) | t5837aep | / | / | / |
| enEp_tentacle | t327aep | 100201881 | cytoplasmic protein NCK2-like | NCK2 [Homo sapiens] XP_016860592.1 |
| | t29023aep | 100206535 | serine/threonine-protein kinase dst1-like | STK17A [Homo sapiens] NP_004751.2 |
| i_female germline2_nurse | t32296aep | 100203304 | hsp70 | HSPA8 [Homo sapiens] NP_006588.1 |
| | t12608aep | 100204298 | fumarate hydratase, mitochondrial-like | FH [Homo sapiens] NP_000134.2 |
| i_female germline1 | t12818aep | 100211523 | ELAV-like protein 3 | ELAVL3 [Homo sapiens] XP_024307178.1 |
| | t9539aep | 100209541 | very-long-chain enoyl-CoA reductase-like | TECR [Homo sapiens] XP_024307558.1 |
| | t24294aep | 100208612 | Uncharacterized | / |
| i_male germline | t4609aep | 100214281 | NADH dehydrogenase [ubiquinone] 1 alpha subcomplex subunit 6-like | NDUFA6 [Homo sapiens] NP_002481.3 |
| | t36963aep | 100215790 | peptidyl-prolyl cis-trans isomerase D-like | PPID [Homo sapiens] NP_005029.1 |
| | t29886aep | | Protein Asterix [Exaiptasia pallida] | / |
| | t14411aep | 100208062 | 26S protease regulatory subunit 7 | PSMC1 [Homo sapiens] NP_002793.2 |
| | t7735aep | 101240495 | uncharacterized protein C10orf35 homolog | FAM241B [Homo sapiens] XP_005269663.1 |
| i_zymogen gland cell | t8678aep | 100192274 | dickkopf-like protein Dlp-2 | DKK1 [Homo sapiens] NP_036374.1 |
| i_spumous mucous | t12601aep | 105850171 | Uncharacterized | macrophage mannose receptor 1-like [Hydra vulgaris] XP_012565955.1 |
| i_zymogen gland cell | t2549aep | 100208683 | Uncharacterized | / |
| | t18337aep | 100208251 | chymotrypsin-like elastase family member 3B | CELA3A [Homo sapiens] NP_005738.4 |
| i_spumous mucous | t16833aep | 101236854 | probable inactive serine/threonine-protein kinase DDB_G0274613 | galaxin-like [Octopus bimaculoides] XP_014775667.1 |
| i_nb3 | t28759aep | 105843076 | protein hunchback-like | / |
| | t31474aep | | / | / |
| | t6076aep | 101235787 | zonadhesin-like | IGFBP2 [Homo sapiens] NP_000588.3 |
| i_nb2 | t36424aep | | universal stress protein A-like protein | / |
| i_nb1 | t9542aep | 100208570 | coiled-coil domain-containing protein 47-like | coiled-coil domain-containing protein 47 [Nematostella vectensis] XP_001635664.1 |
| i_nematocyte | t20281aep | 100201793 | probable Bax inhibitor 1 | TMBIM6 [Homo sapiens] NP_001092046.1 |
| | t26342aep | 100211366 | charged multivesicular body protein 4c-like | CHMP4C [Homo sapiens] NP_689497.1 |
| | t12987aep | 100199474 | protein FAM49B-like | FAM49A [Homo sapiens] NP_110424.1 |
| | t49aep | | predicted protein [Nematostella vectensis] >gi\|156226719\|gb\|EDO47527.1 | / |
| | t14606aep | 100204791 | feline leukemia virus subgroup C receptor-related protein 1-like | FLVCR2 [Homo sapiens] NP_060261.2 |
| i_neuron progenitor | t1704aep | 100212336 | 14-3-3 protein gamma-like | YWHAG [Homo sapiens] NP_036611.2 |
| i_neuron ec | t605aep | | / | |
| | t16657aep | 100213338 | pol-RFamide neuropeptides-like | / |
| | t12724aep | | Gs protein alpha subunit [Tripedalia cystophora] | / |

| | | | | |
|---|---|---|---|---|
| | t11848aep | 100211830 | ubiquilin-1-like | UBQLN1 [Homo sapiens] NP_038466.2 |
| i_neuron/gland progentior | t33270aep | 100209415 | probable serine/threonine-protein kinase DDB_G0268642 | EIF2AK4 [Homo sapiens] NP_001013725.2 |
| | t15889aep | 100203250 | ATPase inhibitor A, mitochondrial-like | bromodomain-containing protein DDB_G0280777-like [Octopus bimaculoides] XP_014776060.1 |
| | t3951aep | 100211845 | histidine--tRNA ligase, cytoplasmic-like | HARS [Homo sapiens] NP_002100.2 |
| | t4973aep | 100207258 | mediator of RNA polymerase II transcription subunit 22-like | MED22 [Homo sapiens] NP_598395.1 |
| | t12704aep | 100210427 | 26S protease regulatory subunit 6A-B-like | PSMC [Homo sapiens] XP_016873515.1 |

**Table S4.** **List of EV transcripts subclustered according to unique cell type expression**

The 5 top genes in each cell cluster are shown

| Gene ID | Gene ID (NCBI) | Gene name | Lineage | Cell type | Anatomical region | Homology (OrthoDB) |
|---|---|---|---|---|---|---|
| t30823aep | / | / | Ectoderm | Ec_basal_disk, Ec_battery_cell, Ec_head, Ec_stem_cell | Head, Body, Foot | / |
| t1568aep | 100197360 | branched-chain-amino-acid aminotransferase, cytosolic-like | Interstitial | i_nb, i_SC, i_stem cell/progenitor | Body | BCAT2 [Homo sapiens] NP_001181.2 |
| t8317aep | / | / | Ectoderm | Ec_basal_disk, Ec_battery_cell, Ec_head, Ec_stem cell | Head, Body, Foot | / |
| t273aep | 105847469 | Uncharacterized | Endoderm, Interstitial | En_tentacle, i_male germline | Tentacle, Body | AF4/FMR2 family member 4-like [Octopus bimaculoides] XP_014773896.1 |
| t1932aep | 100205747 | eukaryotic translation initiation factor 4E-binding protein 3-like | Interstitial | i_female germline, i_SC, i_stem_cell/progenitor | Body | EIF4EBP1 [Homo sapiens] NP_004086.1 |
| t35773aep | 100205436 | soma ferritin-like | Ectoderm, Endoderm, Interstitial | Ec_battery cell, Ec_head, Ec_stem cell, En_tent-nem(pd), i_nematocyte, i_neuron_ec | Head, Body | FTH1 [Homo sapiens] NP_002023.2 |
| t8678aep | 100192274 | Dickkopf 1/2/4-A | Interstitial | i_zimogen gland cell | Body | DKK1 [Homo sapiens] NP_036374.1 |
| t29320aep | / | / | Ectoderm | Ec_battery cell, Ec_head, Ec_stem cell | Head, Body | / |
| t11116aep | 100202036 | actin, non-muscle 6.2-like | Ectoderm | Ec_battery_cell2(mp) | Tentacle | POTEI [Homo sapiens] XP_016860221.1 |
| t15818aep | 101240323 | neuroblast differentiation-associated protein AHNAK | Ectoderm, Endoderm | Ec_basal disk, Ec_battery_cell, Ec_head, Ec_stem_cell, En_tent-nem(pd) | Head, Body, Foot | AHNAK [Homo sapiens] NP_001333375.1 |

**Table S5**. List of top ten EV transcripts matching the scRNA-seq atlas
